# Src kinase phosphorylates Notch1 to inhibit MAML binding

**DOI:** 10.1038/s41598-018-33920-y

**Published:** 2018-10-19

**Authors:** Bryce LaFoya, Jordan A. Munroe, Xinzhu Pu, Allan R. Albig

**Affiliations:** 1 Biomolecular Sciences PhD Program, Boise State University, Boise, ID 83725 USA; 2 Department of Biological Sciences, Boise State University, Boise, ID 83725 USA; 3 Biomolecular Research Center, Boise State University, Boise, ID 83725 USA

## Abstract

Notch signaling is a form of intercellular communication which plays pivotal roles at various stages in development and disease. Previous findings have hinted that integrins and extracellular matrix may regulate Notch signaling, although a mechanistic basis for this interaction had not been identified. Here, we reveal that the regulation of Notch by integrins and extracellular matrix is carried out by Src family kinases (SFKs) working downstream of integrins. We identify a physical interaction between the SFK member, c-Src, and the Notch intracellular domain (NICD) that is enhanced by β3 integrin and the integrin binding ECM protein, MAGP2. Our results demonstrate that c-Src directly phosphorylates the NICD at specific tyrosine residues and that mutation of these phosphorylation sites increases Notch responsive transcriptional activity. Furthermore, we also find that phosphorylation of the NICD by SFKs attenuates Notch mediated transcription by decreasing recruitment of MAML to the Notch co-transcriptional complex. Finally, we also find that SFK activity decreases NICD half-life. Collectively, our results provide important mechanistic data that underlie the emerging role of Notch as a general sensor and responder to extracellular signals.

## Introduction

Canonical Notch signaling is a form of juxtacrine cell communication that affords one cell the ability to induce changes in a neighboring cell’s transcriptome via physical interaction. Notch signaling begins when a Notch ligand from one cell (i.e. the signal sending cell), binds to the transmembrane Notch receptor on an adjacent cell (i.e. the signal receiving cell). Mammals have four different Notch receptors (Notch1–4). Mammalian Notch ligand diversity consists of several transmembrane ligands, including three Delta-like ligands (DLL1, DLL3, and DLL4) and two Jagged ligands (Jag1 and Jag2), as well an assortment of soluble Notch ligands^1,2^. The force applied to the signal receiving cell’s Notch receptor by a neighboring cell’s ligand is critical for Notch activation^3^, and induces a series of proteolytic cleavage events of the Notch receptor, first by ADAM (A Disintegrin and Metalloproteinase) and then by γ-secretase^4,5^. The later severance, known as the S3 cleavage^6^, results in the liberation of the Notch intracellular domain called the NICD fragment. The emancipated NICD fragment translocates to the nucleus where it binds the co-transcription factors Recombining Binding Protein Suppressor of Hairless (RBPJ/CSL), Mastermind-Like (MAML), and the histone acetyltransferase p300/CBP inducing target gene transcription^7,8^. RBPJ associates with chromatin at specific promoter sites, known as CSL sites^9^. It is the Notch-RBPJ interface which forms a groove that recruits the basic domain of MAML, which settles into this furrow as a long α-helix^10^. Assembly of the RBPJ-NICD-MAML ternary complex at CSL sites activates transcription of genes such as, hairy and enhancer of split (*HES*) genes and hairy/enhancer of split related with TYRPW motif (*HEY*) genes^11^.

Being highly versatile and tunable, Notch signaling does not orchestrate cell to cell stimuli exclusively, but rather harmonizes juxtacrine signals with extracellular cues. Recent research has revealed that Notch can respond to a multitude of components within the cellular microenvironment such as hypoxia, hyperglycemia, shear stress, crosstalk with other signaling pathways, and the composition of the extracellular matrix^12^. Previously, we identified a novel signaling axis consisting of the matricellular, integrin binding protein MAGP2 (aka MFAP5), αVβ3 integrin, and Notch1^13^. We showed that ligation of αVβ3 integrin by the RGD containing MAGP2, soluble RGD peptide, and integrin blocking antibodies all were able to regulate the accumulation of the Notch1 intracellular domain (N1ICD) and its transcriptional output^13^. Although we identified a novel mechanism of Notch regulation we were unable to report on the cytosolic events which αVβ3 integrin harnesses in order to coordinate Notch signaling. In this work, we have exposed Src family kinases (SFKs) as modulators of the Notch signaling axis. We reveal that the Notch-SFK interaction is enhanced through β3 integrin and MAGP2. We show that interaction of a SFK member, c-Src, with the Notch intracellular domain leads to tyrosine phosphorylation of the NICD. Furthermore, we identify specific tyrosine residues on the NICD that are phosphorylated by c-Src and are important for NICD transcriptional activity. Finally, we reveal that these tyrosine residues on the NICD serve to decrease NICD-MAML binding. All of this combined reveals mechanistic insight on a novel phosphotyrosine regulatory mechanism of Notch signaling carried out by Src family kinases.

## Results

### Notch1 intracellular domain interacts with c-Src

**Figure 1:**
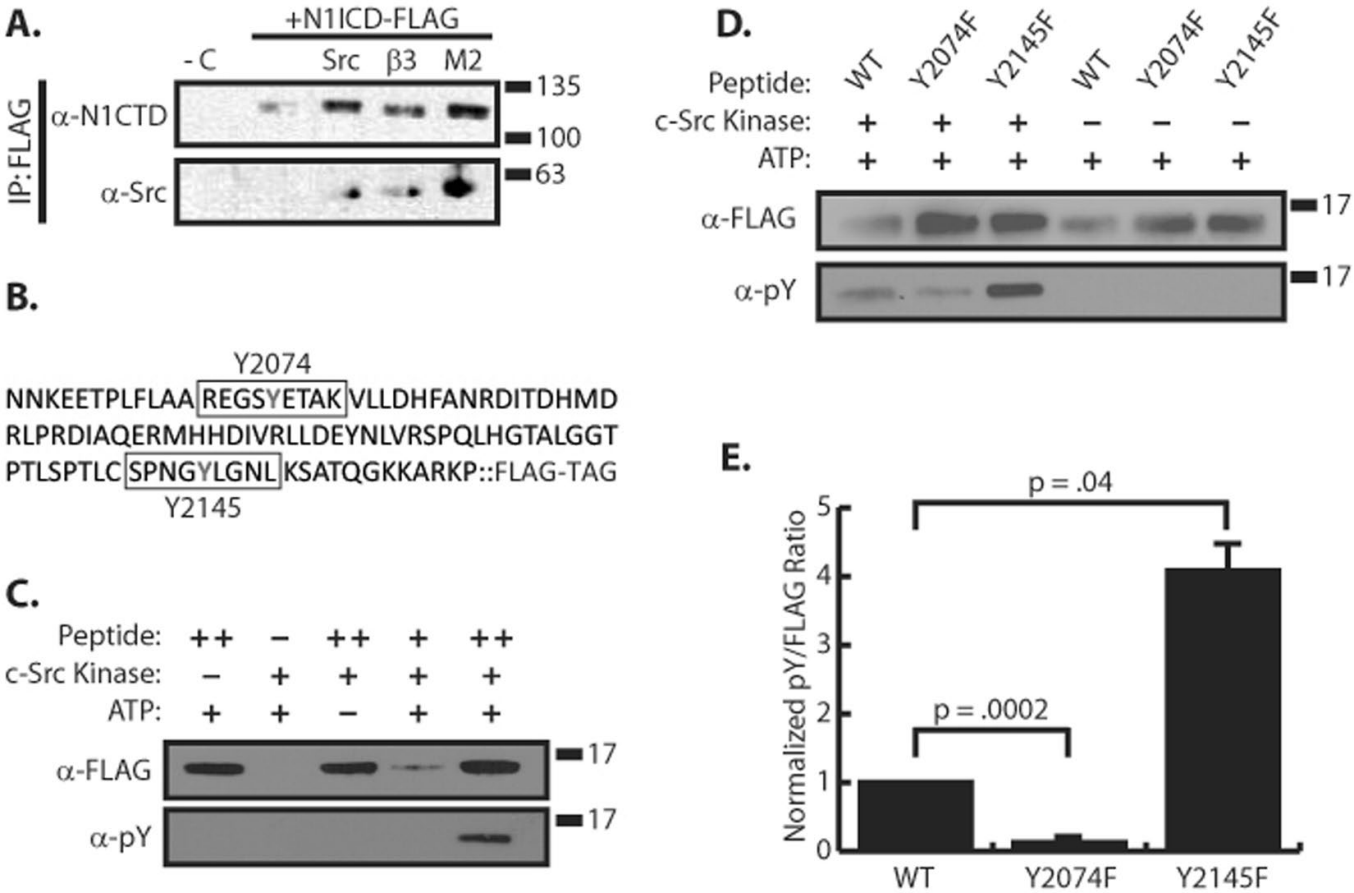
c-Src phosphorylates the N1ICD ankyrin domain. (**A**) Western blot of N1ICD co-immunoprecipitation experiment in 293 T cells. −C denotes a no N1ICD transfection control. Src represents c-Src overexpression. β3 represents β3 integrin overexpression. M2 denotes MAGP2 overexpression. (**B**) Sequence of amino acids 2058–2161 of N1ICD used in subsequent *in vitro* kinase assays. Y2074 and Y2145 are outlined. (**C**) *In vitro* c-Src kinase assay with FLAG tagged peptide containing amino acids 2058–2161 of N1ICD. ++ contains 5X more substrate than+. (**D**) *In vitro c-*Src kinase assay comparing phosphorylation of Y2074F and Y2145F mutant forms of the 2058–2161 peptide. (**E**) Densitometry analysis comparing the level of phosphotyrosine signal from the +Src lanes of panel C combined with multiple identical experiments, n = 4. Normalization was achieved by dividing α-pY by α-FLAG signal. Student’s t-test was performed to determine statistical significance. P-values are reported as p = #. In all panels western blots depict representative images from experiments that were replicated at least three independent times.

Our previous work outlined a connection between Notch signaling and integrin signaling in which integrin activation by MAGP2 serves to inhibit Notch. We wondered what cytosolic mechanism was responsible for this crosstalk between Notch and integrin signaling. A well-known mediator of integrin signaling, c-Src (a Src family kinase member), has been demonstrated to interact with Notch1, and Notch1 has phosphotyrosine residues which can be abated with Src inhibitors^14^. Based on this, we hypothesized that the MAGP2 and αVβ3 integrin enlisted c-Src to carry out Notch regulation. First, we sought to confirm that c-Src physically interacted with N1ICD through co-immunoprecipitation experiments. Due to a lack of commercially available antibodies capable of specifically immunoprecipitating endogenous NICD we opted to use an overexpression model to test our hypothesis. 293 T cells were transfected with cDNA encoding for a 3xFLAG tagged murine N1ICD +/−  cDNA encoding c-Src, and immunoprecipitated with α-FLAG antibodies. Western blot analysis with anti-Src and anti-Notch1 antibodies confirmed a physical N1ICD-Src interaction (Fig. 1A). To determine if c-Src activation downstream of β3 integrin or MAGP2 could facilitate the NICD-Src interaction, we co-transfected 293 T cells with combinations of 3xFLAG-N1ICD and cDNAs encoding β3 integrin or MAGP2. As shown in Fig. 1A, expression of either β3 integrin or MAGP2 drove a N1ICD-Src interaction even in the absence of overexpressed c-Src protein.

### c-Src phosphorylates the ankyrin region of N1ICD

Since Src family members are tyrosine kinases, we wondered whether the N1ICD was phosphorylated by c-Src. Previously, two tyrosine phosphorylation sites at Y2074 and Y2145 were identified on the human N1ICD by immunoprecipitation with anti-pY antibodies and mass spectrometry analysis, although the identity of the kinase responsible for phosphorylation of these residues was not determined^15,16^. We therefore utilized NetPhos 3.1, which predicts phosphorylation sites of eukaryotic proteins^17^, in order to determine if these tyrosine residues might be phosphorylated by c-Src. Y2074 had the highest probability of c-Src phosphorylation, .5 out of 1 while Y2145 phosphorylation was predicted with a score of 0.3 out of 1. Therefore, we sought to verify c-Src mediated phosphorylation using an *in vitro* Src kinase assay. We constructed a C-terminally FLAG tagged 103 amino acid fragment of N1ICD (amino acids 2058–2161) which contains both of the tyrosine sites of interest (Y2074 & Y2145) (Fig. 1B). This peptide, which maps to the C-terminal of the ankyrin domain (ankyrin repeats 6a − 7b + N-terminal of TAD region) was expressed in *E*. *coli*, purified using an anti-FLAG affinity column, and then incubated with purified c-Src kinase in an *in vitro* Src kinase assay. After a 15 minute incubation, samples were subjected to western blot analysis and probed with α-pY (phosphotyrosine) antibodies. We found that c-Src was able to phosphorylate this portion of the N1ICD *in vitro* in an ATP-dependent manner (Fig. 1C). Seeking to identify which residues (Y2074, Y2145, or both) became phosphorylated we created two more peptides in which we substituted tyrosine for phenylalanine (Y → F) at these sites and submitted these proteins to our *in vitro* c-Src kinase assay (Fig. 1D). We found that the Y2074F mutant was not as heavily tyrosine phosphorylated when compared to the wild type (WT) peptide, thus pointing to this being an important residue for c-Src phosphorylation. Although, the Y2074F mutant did display some tyrosine phosphorylation, suggesting that while Y2074 was still a likely candidate of phosphorylation, c-Src must be phosphorylating at least one other site on the peptide. Surprisingly, Y2145F mutant had enhanced tyrosine phosphorylation when compared to WT peptide. Subsequent densitometry analysis revealed that c-Src phosphorylated the WT N1ICD ~10 times greater than the Y2074F N1ICD, whereas the Y2145F N1ICD had ~four times more phosphorylation than the WT (Fig. 1E). These results confirm that c-Src can phosphorylate at least two sites on the N1ICD.

**Figure 2:**
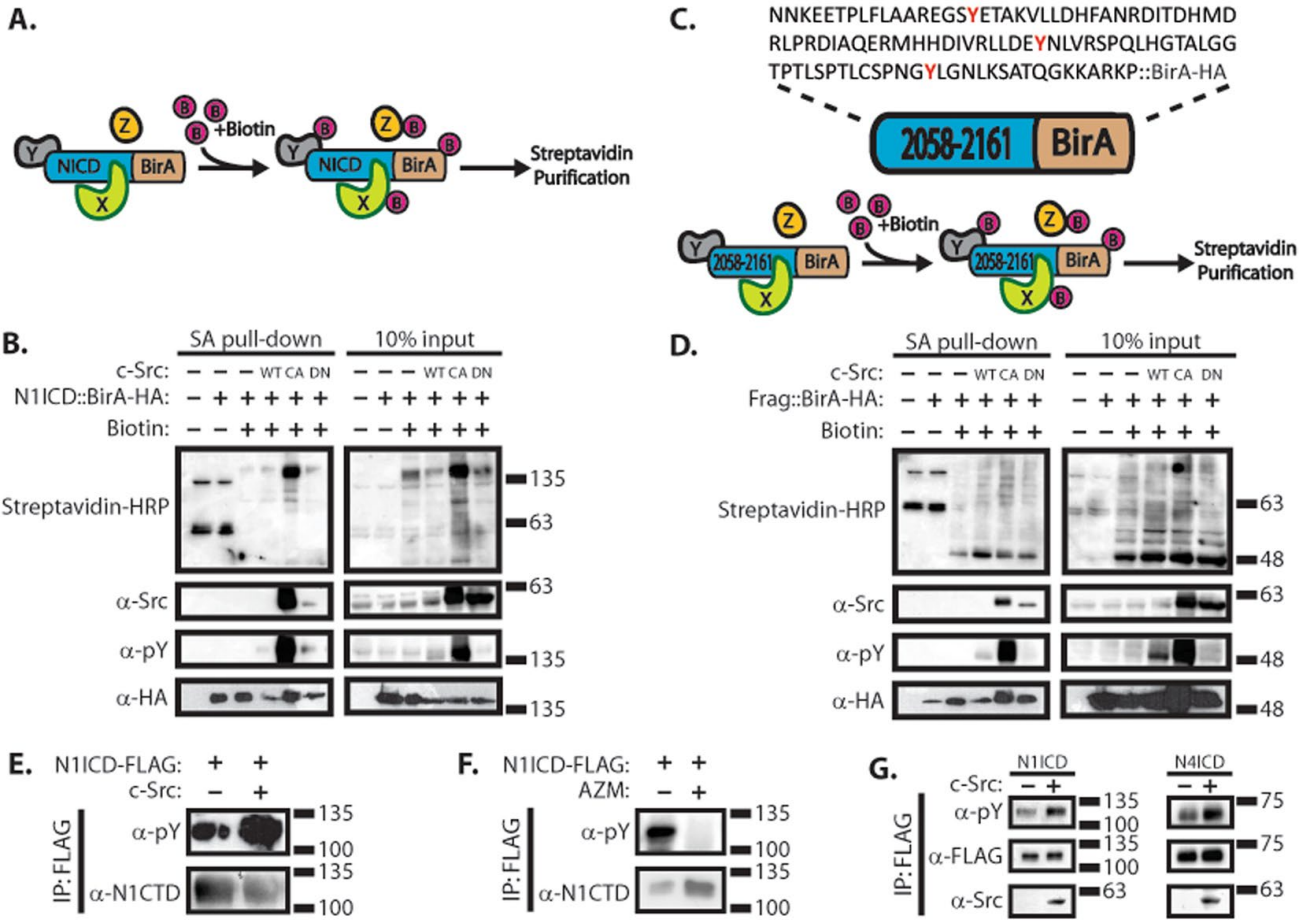
Phosphorylation of tyrosine residues on the N1ICD are SFK dependent. (**A**) Diagram of BioID experiment. In the presence of biotin, the NICD::BirA fusion protein biotinylates nearby proteins which can be affinity captured through streptavidin purification. (**B**) Western blot analysis of affinity captured material from BioID experiment using a N1ICD::BirA-HA fusion protein in 293 T cells. Expression of Src variants is denoted as WT = wild-type, CA = constitutively active, and DN = dominant negative. (**C**) Amino acid sequence of 2058–2161::BirA-HA fusion protein containing a 104 amino acid fragment of N1ICD fused to a biotin ligase. (**D**) Western blot analysis of affinity captured material from BioID experiment using the 2058–2161::BirA-HA fusion in 293 T cells. (**E**) Western blot analysis of immunoprecipitated N1ICD in the presence or absence of c-Src overexpression in 293 T cells. (**F**) Western blot analysis of immunoprecipitated N1ICD under conditions SFK inhibition (AZM475271) and control in 293 T cells. (**G**) Western blot analysis of immunoprecipitated N1ICD and N4ICD in the presence or absence of c-Src overexpression in 293 T cells. In all panels, western blots depict representative images from experiments that were replicated at least three independent times.

While these results demonstrated that c-Src can phosphorylate the 2058–2161 fragment of N1ICD *in vitro*, it was important to determine if this region was sufficient for c-Src interaction and phosphorylation *in vivo* where this fragment would be expected to be more properly folded. To test this, we made fusion proteins of the full-length N1ICD domain and the 2058–2161 fragment fused to HA tagged BirA biotin ligase, in order to perform proximity biotin ligation (i.e. BioID^18^) experiments (Fig. 2B,D). In these experiments, the BirA enzyme biotinylates proteins which are within close proximity (20–30 nm) to the fusion protein (Fig. 2A,C). In addition, we co-expressed wild type (WT), constitutively active (CA), and dominant negative (DN) forms of Src kinase in combination with our fusion proteins. Streptavidin pulldowns were performed to affinity capture biotinylated proteins, and streptavidin-HRP was used to detect biotinylated species by western blot. Subsequent blotting with α-Src primary antibodies confirmed Src interacts with, or at least is within close proximity to both the N1ICD::BirA and 2058–2161::BirA fusions. Furthermore, upon overexpression of WT and CA Src, both fusions were found to be tyrosine phosphorylated. Additionally, overexpression of DN Src induced no such phosphorylation in the 2058–2161::BirA fusion. These results suggest the 2058–2161 region which maps to the C-terminal of the ankyrin domain of the N1ICD is sufficient for Src interaction.

### N1ICD is phosphorylated in a SFK-dependent manner

Having established that SFKs interact with and phosphorylate the intracellular domain of Notch, it was important to determine whether SFKs alone are responsible for NICD phosphorylation, or if other tyrosine kinases phosphorylate NICD. To this end, we expressed 3xFLAG N1ICD in 293 T cells in the presence or absence of c-Src overexpression and immunoprecipitated the N1ICD using α-FLAG antibodies. Western blot analysis reveals that c-Src overexpression induces greater pY signal emanating from N1ICD compared to the no c-Src overexpression control (Fig. 2E). To investigate whether SFK inhibition altered N1ICD phosphorylation, N1ICD pulldowns were performed after 24 hour treatment of a highly specific inhibitor of SFKs (AZM475271)^19^, and it was found that SFK activity is required for tyrosine phosphorylation of N1ICD (Fig. 2F). Taken together, this evidence suggests that phosphorylation of tyrosine residues on the N1ICD occurs in a SFK-dependent fashion. Finally, we wanted to see whether this phenomenon is specific to Notch1. We expressed FLAG tagged N1ICD and N4ICD in the presence or absence of c-Src overexpression in 293 T cells and immunoprecipitated the NICDs using α-FLAG antibodies (Fig. 2G). Western blot analysis of the immunoprecipitated material revealed that both NICD molecules immunoprecipitated with c-Src under conditions of c-Src overexpression. Furthermore, N4ICD was also tyrosine phosphorylated and this was enhanced through c-Src overexpression. Thus, the interaction between c-Src and the Notch intracellular domain is not specific to Notch1, but rather a broader mechanism which encompasses Notch4 as well.

**Figure 3:**
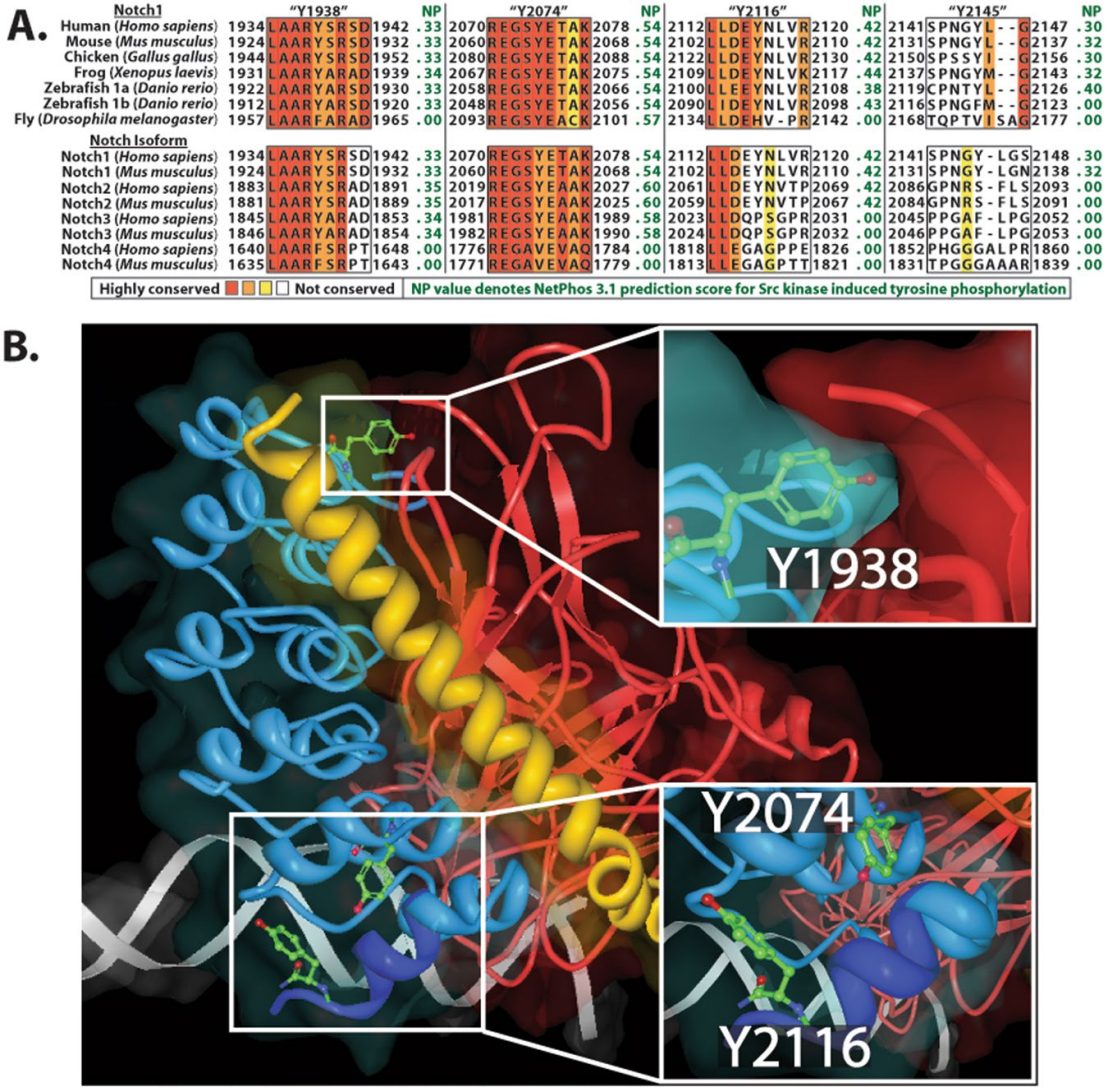
Sequence alignment and crystal structure analysis of NICD phosphotyrosine sites. (**A**) Sequence alignment of four tyrosine sites within the Notch intracellular domain comparing conservation of sites across different species and different Notch isoforms. Red = highly conserved, white = not conserved. NP denotes NetPhos 3.1 prediction scores for likelihood of c-Src phosphorylation of the sequence. (**B**) Partial crystal structure analysis of Notch ternary transcriptional complex. N1ICD ankyrin domain = blue (N-terminal of TAD = dark blue), MAML basic domain = yellow, RBPJ = red. Insets depict where Y1928, Y2074, and Y2116 are found in the N1ICD ankyrin domain.

It was important to map tyrosine residues that were phosphorylated by SFKs. To accomplish this, we first submitted our *in vitro* c-Src kinase assays samples (Fig. 1, and Supplementary Fig. 1) to mass spectrometry analysis. No phosphortyrosine residues were detected in samples which were not incubated with c-Src. Only in c-Src incubated samples were phosphotyrosines detected at the Y2074 (Y2064 in mice) and Y2145 (Y2135 in mice) as predicted by our NetPhos 3.1 analysis. In addition, we were able to identify phosphotyrosine at Y2116 (Y2106 in mice) which had previously escaped our initial attention. To determine if these sites are phosphorylated in living cells, 3xFLAG N1ICD was transfected into 293 T cells, then immunoprecipitated using α-FLAG antibodies. Mass spectrometry analysis of these samples verified phosphorylation at Y2074, Y2116, and Y2145 and also identified an additional phosphorylated residue, Y1938 (Y1928 in mice), which is not present on our 2058–2161 peptide (Supplementary Fig. 2). To examine the conservation of these sites, we performed a sequence alignment analysis of the identified phosphorylation sites across several species and across all four Notch isoforms in humans and mice (Fig. 3A). For our analysis we also included the NetPhos 3.1 score which predicts the likelihood of c-Src induced tyrosine phosphorylation at each of these sites. This analysis revealed that Y1938 and Y2074 are highly conserved, Y2116 is moderately conserved, and Y2145 is somewhat conserved. It should be noted, that residues which lie within the highly structured ankyrin domain (Y1938, Y2074, and Y2116) are more conserved than Y2145 which lies proximal to the ankyrin domain. The position of residues Y1938, Y2074, and Y2116 within the N1ICD/RBPJ/MAML co-crystal (PDB# 3NBN) are indicated in Fig. 3B.

### SFKs regulate Notch1 transcriptional activity

**Figure 4:**
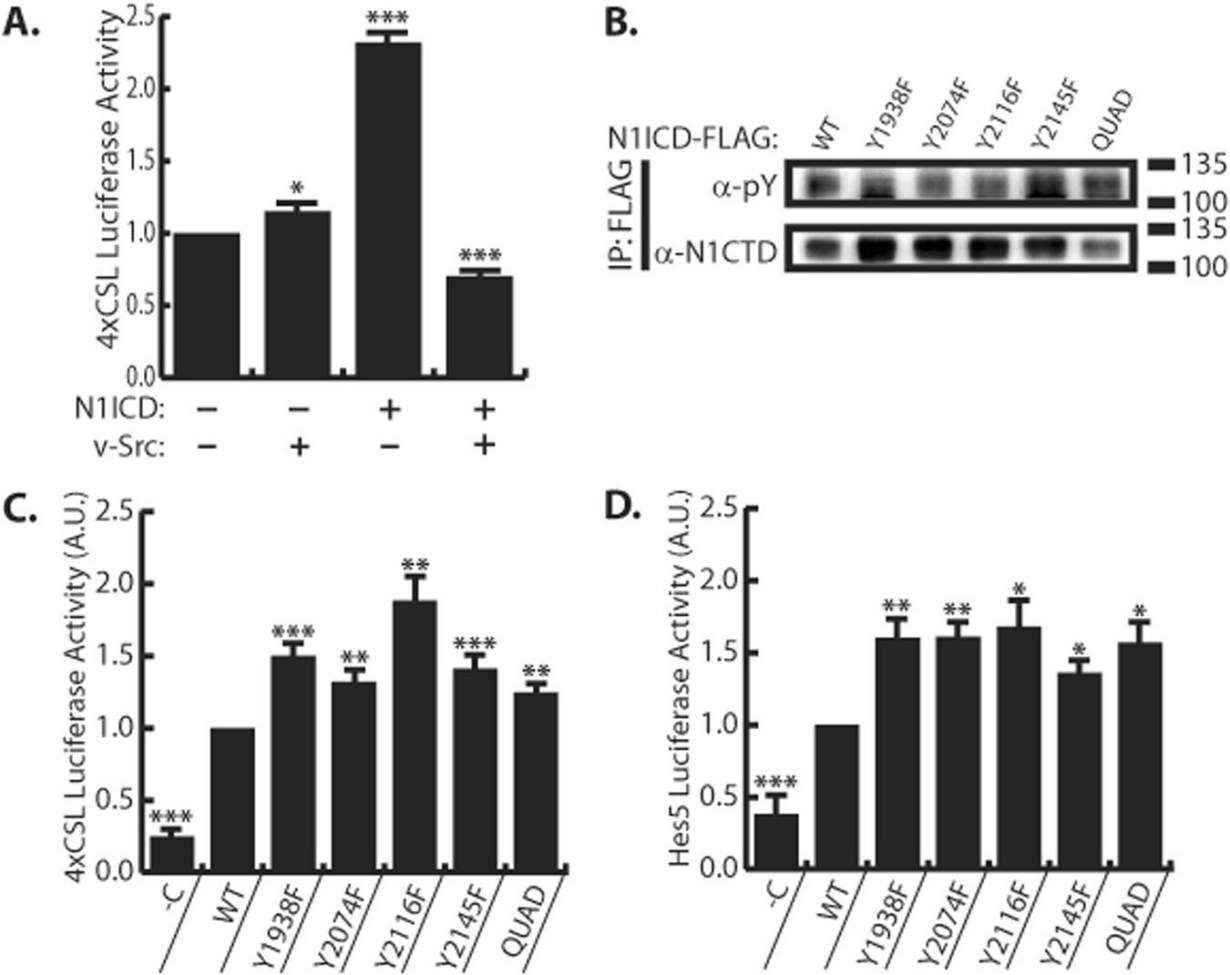
SFKs reduce N1ICD transcriptional activity. (**A**) 4xCSL luciferase assay in 293 T cells in the presence or absence N1ICD overexpression or v-Src overexpression, n = 6. (**B**) Anti-phosphotyrosine western blot analysis of immunoprecipitated N1ICD tyrosine mutants in 293 T cells. “Quad” denotes a Y1938F, Y2074F, Y2116F, Y2145F mutant N1ICD. Image is a representative image from experiments that were replicated three independent times (**C**) 4xCSL luciferase assay in 293 T cells expressing WT and mutant N1ICDs, n = 16. −C denotes non-N1ICD transfected control. (**D**) Hes5 luciferase assay in 293 T cells expressing WT and mutant N1ICDs, n = 7. −C denotes non-N1ICD transfected control. For A, student’s t-test was performed to determine stastical significance compared to -N1ICD/-vSrc control. For C-D, student’s t-test was performed to determine stastical significance compared to WT control. P-values are reported as * <0.05, ** <0.01, *** <0.001. In all panels, western blots depict representative images from experiments that were replicated at least three independent times.

Having established a physical interaction between N1ICD and SFKs, it was important to determine if SFKs control Notch transcriptional activity. To accomplish this, 293 T cells were transfected with Notch responsive 4xCSL reporter which expresses luciferase in response to activation of Notch signaling. Surprisingly, in the absence of overexpressed N1ICD, v-Src had very little effect on Notch activity. However, cells co-transfected with N1ICD and v-Src demonstrated decreased luciferase activity compared to cells transfect with N1ICD alone (Fig. 4A). This result suggests that v-Src activity downregulates Notch mediated transcription. Having found and confirmed sites of SFK mediated tyrosine phosphorylation on the N1ICD, it was important to determine if phosphorylation at these sites was important for Notch transcriptional activity. To this end, we introduced Y → F substitutions at Y1938, Y2074, Y2116, and Y2145 within our 3xFLAG N1ICD construct. We also constructed a “Quad” mutant N1ICD which has Y1938F, Y2074F, Y2116F, and Y2145F substitutions. We expressed these mutant forms of N1ICD in 293 T cells and immunoprecipitated them using α-FLAG antibodies. To our surprise, we did not detect an obvious correlation between Y → F mutation and anti-pY western blot signal (Fig. 4B). Nonetheless, all of the Y → F N1ICD mutants demonstrated enhanced transcriptional activity on the 4xCSL and Hes5 promoters as compared to WT N1ICD (Fig. 4C,D). To summarize these findings, enhanced SFK activity (i.e. v-Src expression) decreases Notch transcriptional output, while removal of sites of tyrosine phosphorylation increases Notch transcriptional output. Taken together, these results suggest that SFK activity impedes Notch target gene transcription.

### SFK phosphorylation sites decrease NICD-MAML binding

**Figure 5:**
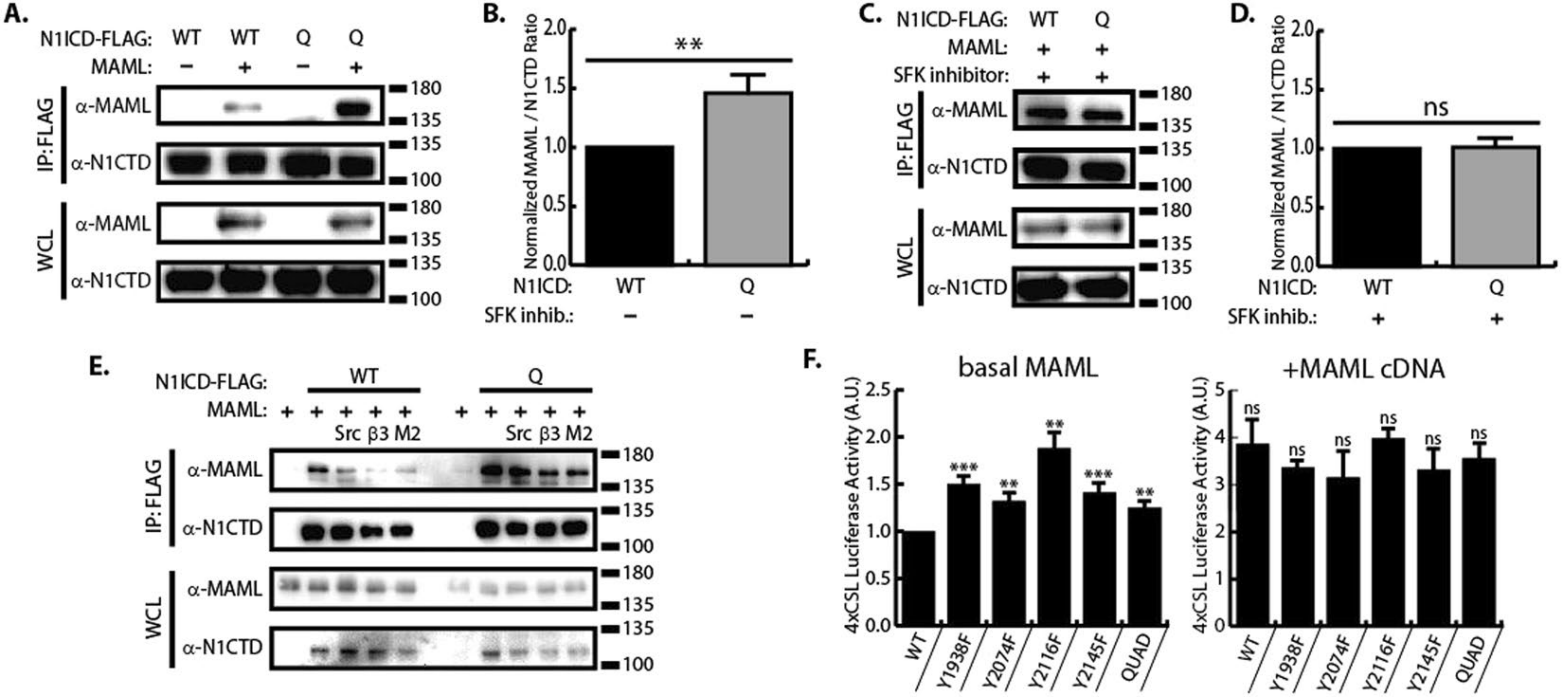
N1ICD tyrosine mutants display enhanced MAML binding. (**A**) Western blot analysis of N1ICD/MAML co-immunoprecipitations comparing WT N1ICD and Quad N1ICD in 293 T cells. (**B**) Densitometry comparing levels of MAML co-immunoprecipitation with WT vs Quad N1ICD under conditions of normal SFK activity (n = 4). (**C**) Western blot analysis of N1ICD/MAML co-immunoprecipitations comparing WT N1ICD and Quad N1ICD under condition of SFK inhibition (AZM475271) in 293 T cells. (**D**) Densitometry comparing levels of MAML co-immunoprecipitation with WT vs Quad N1ICD under conditions of SFK inhibition (n = 3). (**E**) Western blot analysis of N1ICD/MAML co-immunoprecipitations in the presence of c-Src, β3 integrin, or MAGP2 (M2) in 293 T cells, (n = 2). (**F**) 4xCSL luciferase assay comparing transcriptional activity of N1ICD tyrosine mutants in the presence or absence of MAML overexpression in 293 T cells, n = 8. In all panels, western blots depict representative images from experiments that were replicated at least three independent times. Where applicable, student’s t-test was performed to determine statistical significance. P-values are reported as * < 0.05, ** < 0.01, *** < 0.001, ns > 0.05.

Based on the crystal structure analysis of the Notch ternary complex, we sought to assess whether SFKs play a role in Notch ternary complex formation. To test this, we expressed FLAG tagged N1ICD Quad mutant or WT N1ICD in 293 T cells in the presence or absence of transfected MAML1 cDNA and used anti-FLAG co-immunoprecipitation to monitor N1ICD-MAML interactions. As shown in Fig. 5A,B, the Quad mutant co-immunoprecipitated more MAML1 than the WT N1ICD. We then hypothesized that inhibition of SFK activity would equilibrate the amount of MAML interaction between WT and Quad N1ICD forms. To test this, we performed the same co-immunoprecipitation, but this time in the presence of SFK inhibitor. Under conditions of SFK inhibition both WT N1ICD and Quad N1ICD co-immunoprecipitate equal amounts of MAML1 (Fig. 5C,D). In Fig. 1A, we demonstrated that Src-N1ICD interaction was strengthened by overexpression of β3 integrin or MAGP2, therefore we sought to determine if β3 integrin or MAGP2 may also regulate N1ICD-MAML interaction. To test this, we examined the N1ICD-MAML interaction by co-immunoprecipitation in the presence of β3 integrin or MAGP2 expression. As shown in Fig. 5E, overexpression of integrin machinery including c-Src, β3 integrin, or MAGP2 all decreased WT N1ICD-MAML interaction while the Quad N1ICD mutant was resistant to this effect. Taken together, these results suggest that SFK phosphorylation of N1ICD decreases N1ICD-MAML binding.

Having confirmed that SFKs are important for N1ICD-MAML interaction, we next sought to determine if the increased transcriptional potency of the N1ICD tyrosine mutants was due to enhanced MAML recruitment. We hypothesized that the enhanced transcriptional potency of the N1ICD tyrosine mutants was due to their enhanced ability to recruit MAML from a limited endogenous supply. We therefore reasoned that providing a surplus of MAML (i.e. overexpression) would elevate WT N1ICD transcriptional output to match that of the N1ICD tyrosine mutants. To test this, we compared the ability of the WT and tyrosine mutant N1ICD forms to drive transcription from the 4xCSL luciferase reporter assay in the presence or absence of MAML cDNA (Fig. 5F). In contrast to conditions of basal MAML expression where tyrosine mutant N1ICD forms display enhanced transcriptional potency, during MAML overexpression the WT and all five tyrosine mutant forms of N1ICD had equivalent transcriptional potency (Fig. 5F). These results suggest that MAML is the rate limiting factor during Notch target gene transcription in this system, because when MAML is in surplus (during overexpression), WT and tyrosine mutant N1ICDs have equilibrated transcriptional activity. Taken together, this evidence suggests SFK phosphorylation sites within the N1ICD serve to impede N1ICD recruitment of MAML, thus decreasing Notch transcriptional output.

### SFK activity decreases N1ICD half-life

**Figure 6:**
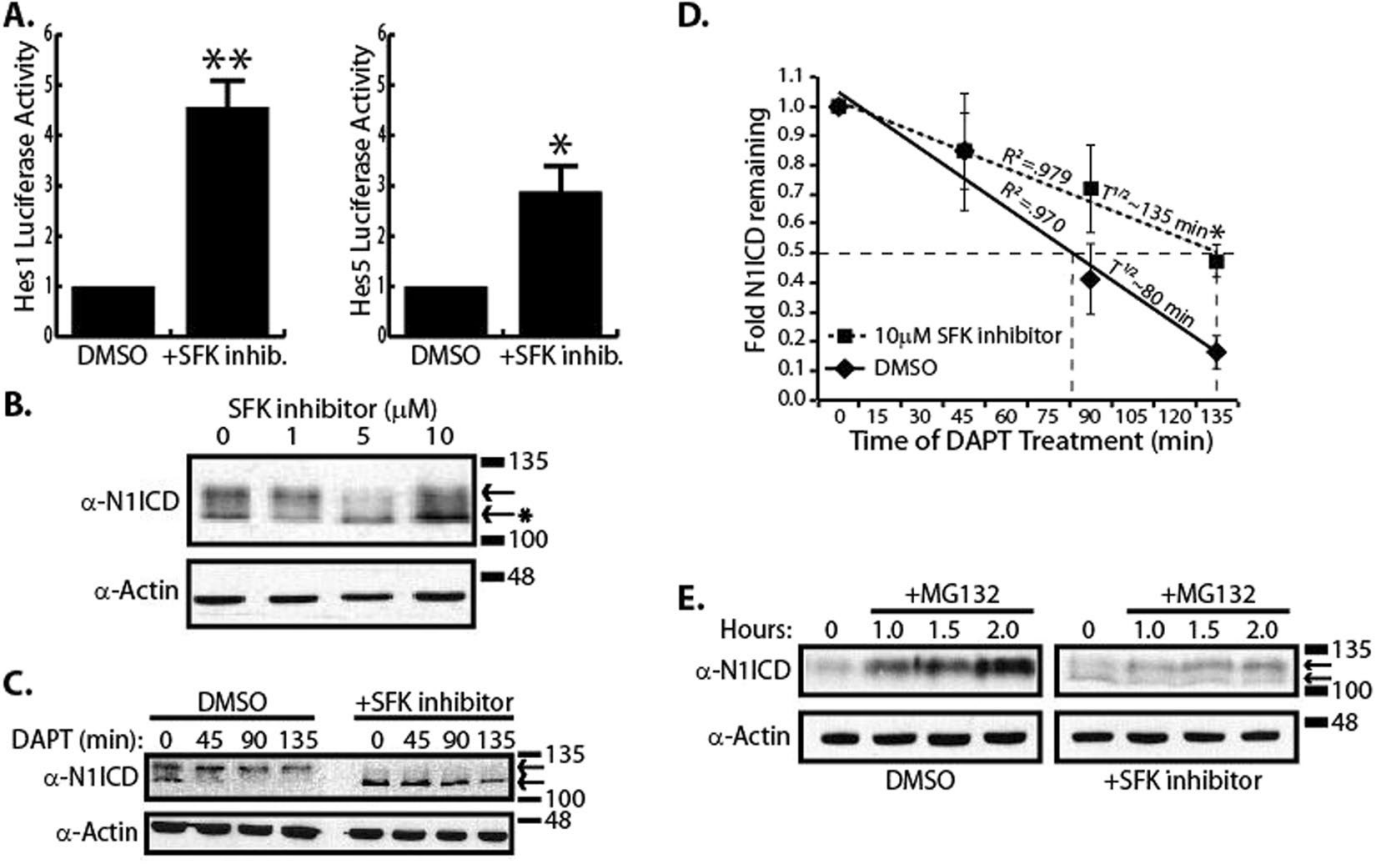
SFKs decreases the N1ICD half-life. (**A**) Hes1 and Hes5 luciferase assay in HMEC-1 cells in the presence or absence of SFK inhibitor (AZM475271), n = 4. (**B**) Western blot of HMEC-1 lysates under conditions of increasing concentrations of SFK inhibitor (AZM475271). The arrows denote two distinct N1ICD species. The asterisk points out that SFK inhibition leads to accumulation of a higher mobility N1ICD species. (**C**) Western blot analysis of N1ICD half-life under conditions of SFK inhibition and DMSO control in HMEC-1 cells. The arrows denote two distinct N1ICD species. (**D**) Densitometry and half-life analysis of combined upper and lower N1ICD western blot bands, normalized to Actin, from panel C. Data represents n = 4 independent experiments and * indicates p < 0.05, students t-test. (**E**) Western blot analysis of N1ICD accumulation under condition of proteasomal inhibition using MG132 in HMEC-1 cells.

Since Notch is heavily involved in many developmental processes such as angiogenesis^20,21^, and MAGP2 coordinates Notch signaling in order to stimulate angiogenesis^22^, we wanted to establish whether SFKs alter Notch activity in endothelial cells. To accomplish this, human microvascular endothelial cells (HMEC-1) were transfected with Notch responsive Hes1 and Hes5 luciferase reporters and treated with the SFK inhibitor, AZM (AZM475271). As shown in Fig. 6A, both the Hes1 and Hes5 promoters demonstrated enhanced transcriptional activity under conditions of SFK inhibition compared to DMSO control. Since transcription from Notch responsive promoters is dependent on N1ICD, we next determined if inhibition of SFKs also increased the amount of N1ICD in HMEC cells. HMEC cells were treated with increasing concentrations of AZM and N1ICD was detected in whole cell lysates by western blot analysis with anti-VAL1744 antibodies which only recognize N1ICD after S3 cleavage. As shown in Fig. 6B, N1ICD appeared as a doublet when blotting HMEC-1 cell lysates with α-N1ICD antibodies, and it was noticed that after SFK inhibition, a larger proportion of N1ICD migrated as a low molecular weight species. We estimated that the lower N1ICD band migrated ~9 kDa faster than the upper band.

Ubiquitin is a 9 kDa protein^23^ that is attached to proteins marking them for proteasomal degradation^24^. Since the two N1ICD species migrated with a difference of ~9 kDa, we hypothesized that the higher molecular weight species might be ubiquitinated and that SFKs may regulate Notch signaling through manipulation of NICD half-life. In order to track N1ICD half-life, HMEC-1 cells were treated with DAPT (10 µM) to inhibit γ-secretase activity and the amount of remaining N1ICD was monitored by western blot 0, 45, 90, 135 minutes after DAPT treatment. Interestingly, it was found that before DAPT treatment a N1ICD doublet was produced, but following DAPT treatment the lower molecular weight species disappeared with time before the higher molecular weight species. Performing this same experiment in the presence of SFK inhibition resulted in a majority of the N1ICD species to run as the lower molecular weight band (Fig. 6C) that did not accumulate as a higher molecular weight band. Densitometry of both higher and lower molecular weight species showed that N1ICD had a half-life of ~80 minutes which was lengthened to ~135 minutes in the presence of SFK inhibitor (Fig. 6D). Since N1ICD is degraded by the proteasome and accumulates with treatment of the proteasome inhibitor, MG132^25^, we hypothesized that SFKs normally decrease N1ICD stability by inducing ubiquitination of N1ICD. To test this, we treated HMEC-1 cells overnight with DMSO or SFK inhibitor then with MG132 for 0–2 hours and monitored N1ICD accumulation by western blot (Fig. 6E). In support of our hypothesis, it was found that the higher molecular weight species accumulated upon proteasomal disruption. We noted that the higher mobility fragment accumulates rapidly under control conditions, but this accumulation is delayed through inhibition of SFKs. Despite these observations however, we were unable to successfully detect ubiquitin on N1ICD by western blot under any condition and therefore, the biochemical nature of the observed band shift and half-life affect remain unknown. Taken together, these results reveal that SFK inhibition reduced the accumulation, whereas proteasomal inhibition enhanced accumulation, of the higher molecular weight N1ICD in HMEC-1 cells.

## Discussion

The Notch signaling mechanism has long been known to facilitate communication between adjacent cells thus allowing cells to sense, and respond, to their immediate neighbors in the cellular microenvironment^26^. However, sensing and responding to cell-cell interactions appears to be only a part of the larger emerging function of Notch. Indeed, Notch has shown the ability to act as a general sensor for diverse signals in the cellular microenvironment including growth factors, extracellular matrix, hyperglycemia, hypoxia, and shear stress^12^.

**Figure 7:**
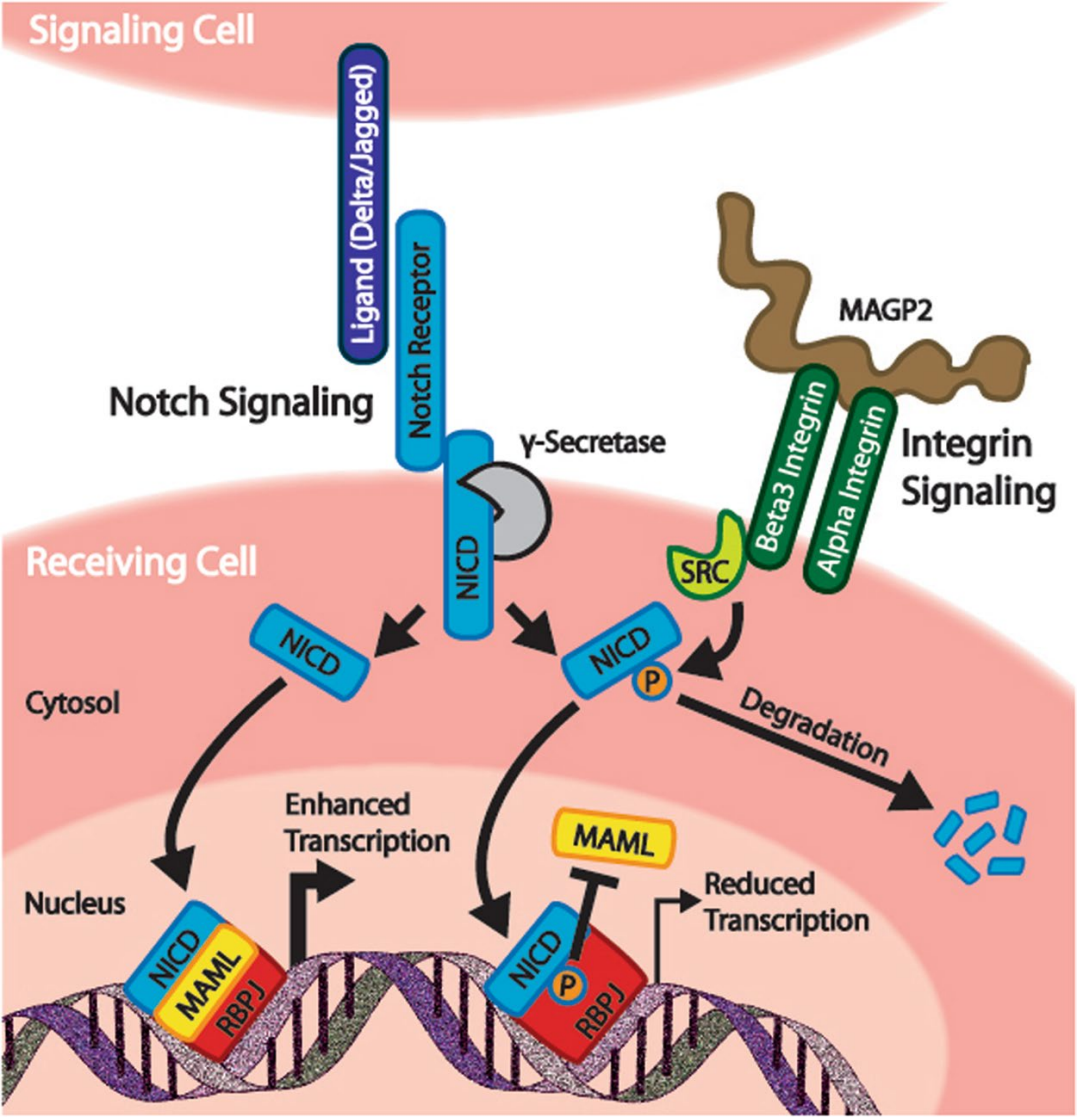
SFK regulation of the Notch signaling pathway. Proposed mechanism of the regulation of Notch signaling by Src family kinases (SFKs). Integrin-induced SFK activity serves to phosphorylate the Notch intracellular domain. This phosphorylation leads to decreased recruitment of MAML to the Notch transcriptional complex and subsequent reduction in target gene transcription. Phosphorylation of the Notch intracellular domain may lead to reduced half-life of the protein.

The understanding that extracellular matrix (ECM) is capable of regulating Notch has been known for some time and several mechanisms for this have been described. For instance, basement membrane laminins regulate expression of the Notch ligand DLL4 thereby restricting tip cell development in branching endothelial cells^27,28^. Other ECM proteins including collagen IV^29^, CCN3^30^, and YB-1^31^ regulate Notch through direct interactions with Notch receptors. Finally, our previous work determined that the ECM proteins MAGP2 and EGFL7 control Notch through RGD-dependent integrin binding, but did not describe the molecular mechanism through which this was accomplished^13,22^. Our current results build upon our previous work by now describing a signaling mechanism that couples ECM proteins to Notch through an integrin/SFK signaling circuit. We observed that c-Src interacts with the intracellular domain of Notch1, and this interaction is enhanced through β3 integrin or MAGP2 expression (Fig. 1A Furthermore, we have identified four tyrosine residues (Y1938, Y2074, Y2116, and Y2145) on the N1ICD which serve as SFK substrates (Figs 1–3). We demonstrated that removal of these SFK phosphorylation sites leads to enhanced transcriptional activity and MAML binding (Figs 4C,D and 5)). We also found that N1ICD stability is regulated by SFK activity (Fig. 6C–E). Based on this evidence, we propose a regulatory mechanism whereby integrin activation of Src family kinases drives phosphorylation of N1ICD to impede the Notch signaling pathway by decreasing N1ICD-MAML interactions (Fig. 7). In addition, SFK induced phosphorylation serves to reduce N1ICD half-life, possibly by targeting the N1ICD for proteasomal degradation (Fig. 7). Interestingly, our results are not the first to link Notch to integrin signaling. Rather, Mo *et al*., previously determined that Integrin-linked kinase (ILK) directly phosphorylates NICD to recruit the ubiquitin ligase FBW7 and ultimately destabilize the N1ICD protein^32^. Collectively, our findings greatly enhance our molecular understanding of how Notch activity is modulated by extracellular matrix within the cellular microenvironment.

The tyrosine residues we analyzed in this study are found within or in close proximity to the Notch ankyrin domain, and their positions offer clues as to the biological function of tyrosine phosphorylation at these sites. Y1938 is located between alpha-helices 2a and 2b of the ankyrin domain^33^ and is highly conserved in vertebrate Notch 1–3 proteins (Fig. 3A). The conservation of this site suggests an important role in Notch signaling which, given the projection towards RBPJ, may be to regulate RBPJ-NICD interactions (Fig. 3B). Y2074 is located in alpha-helix 6a, while Y2116 is located just C-terminal to alpha-helix 7b^33^. Y2074 is conserved in Notch1 of all species examined as well as human and mouse Notch 1–3 proteins. Y2116 is conserved in vertebrates as well as human and mouse Notch 1–2 proteins (Fig. 3A). These residues either project towards (Y2074) or are within (Y2116) the transactivation (TAD) domain (Fig. 3B). This region of the TAD domain is required for recruitment of p300 acetyltransferase to the N1ICD transcriptional complex and thus phosphorylation at these sites may regulate p300 interaction^33,34^. In addition, the seventh ankyrin repeat has been implicated in controlling the stability and folding of the entire ankyrin domain^35^ and it is tempting to speculate that phosphorylation of Y2074 and/or Y2116 may affect the overall structure of the ankyrin domain. Finally, the Y2145 residue is also conserved in vertebrates, but is only found in Notch1. Y2145 is not located within the existing crystal structures of N1ICD, but maps to a position just N-terminal to the second nuclear localization signal. Our transcriptional data determined that Y → F mutation at any of these residues enhanced Notch transcriptional activity, suggesting that phosphorylation at these sites represses Notch mediated transcription (Figs. 4C and 4D). In support of this, Notch mediated transcription was enhanced in the presence of SFK inhibitors and repressed in the presence of constitutively active v-Src (Figs 4A and 6A). We also observed that simultaneous Y → F mutation of all four tyrosine residues (i.e. Quad mutant) resulted in increased N1ICD binding to MAML, and that this was nullified by treatment with SFK inhibitor (Fig. 5A–D).

Several lines of evidence are now converging to suggest that the TAD domain is an important regulatory site for controlling Notch activity. The N-terminal region of the TAD domain, overlaps with ankyrin repeat 7b at the C-terminal of the ankyrin domain, and is defined as a region of NICD critical for p300 interaction^34^. This region of NICD has also been proposed to be at the N1ICD/MAML interface^10^. Our data demonstrate that MAML interaction with N1ICD is enhanced by Y → F mutations in this region and decreased by c-Src overexpression (Fig. 5A–E). Similarly, previous results also found that Nemo-like kinase (NLK) phosphorylates serine residues in this region (S2121 and S2141) resulting in reduced MAML interaction^36^. Together, these results suggest that tyrosine and/or serine phosphorylation within the TAD domain may decrease MAML interaction with N1ICD, an idea that is supported by our data showing that overexpression of MAML equilibrates mutant N1ICD transcriptional activity to WT levels (Fig. 5F). An additional point of NICD regulation that falls within the TAD domain is ILK mediated phosphorylation of human N1ICD at S2184 which recruits FBW7 and drives ubiquitination of NICD^32^. Finally, our results showing that Y2145F mutation resulted in increased phosphorylation *in vitro* (Fig. 1D,E) hint that phosphorylation at Y2145 may suppress phosphorylation at other NICD sites thus functioning as a regulatory site for Notch signaling, although this idea will need additional verification in future studies. Collectively, the fact that SFKs, NLK, and ILK all influence Notch signaling through phosphorylation with the NICD TAD domains, support a central role for this domain in controlling Notch signaling output.

Broader implications of our findings have yet to be determined. However, the idea that Notch signaling can be influenced by integrins and SFKs may have broad reaching implications for how Notch functions within cellular microenvironments. For instance, SFK signaling is not restricted to integrins but rather, SFKs are activated by a wide range of other signaling mechanisms such as RTK and GPCR receptors^37^. Therefore, an important research goal will be to determine if these signaling mechanisms engage in crosstalk with Notch through SFK mediated NICD phosphorylation. In addition, Notch and integrins are highly conserved and function in a wide variety of biological circumstances. It will be important to discover how the interplay between integrins and Notch function in this wide range of biological situations. Finally, based on our sequence comparison of N1-N4ICD (Fig. 3A), it is clear that not all of the tyrosine sites we identified are conserved in all Notch NICDs. Based on this observation, an interesting possibility is that SFKs may be able to differentially regulate various Notch isoforms and establish alternative Notch signaling activities thereby diversifying overall Notch output.

In summary, this work continues to build on the growing evidence that Notch is more than a cell-cell signaling mechanism, but rather, an integrator of multiple cues within the cellular microenvironment. This work adds an important piece to this puzzle by providing mechanistic insight about how extracellular matrix composition regulates Notch via integrins and Src family kinase signaling. Future work will continue to dissect additional molecular mechanisms by which Notch senses and responds to stimuli from the cellular microenvironment.

## Materials and Methods

### Antibodies

For western blotting primary antibodies against cleaved Notch1 (N1ICD) (Val1744, #2421), phosphotyrosine (pY) (P-Tyr-100, #9411), Src (32G6), and MAML1 (MAML1, #4608) were purchased from Cell Signaling Technology. Primary antibodies against β-actin (sc-47778), HA tag (sc-57592), Notch1 C-terminal domain (N1CTD) (sc-6014-R), and vinculin (sc-5573) were purchased from Santa Cruz Biotechnology. Primary antibodies against FLAG (DYKDDDDK) tag (A00170) were purchased from GenScript. Secondary antibodies consisted of horseradish peroxidase conjugated antibodies α-mouse (NA931V) and α-rabbit (NA934V) purchased from GE Healthcare Life Sciences.

### Cell culture

HMEC-1 cells were cultured in MCDB 131 media supplemented with 10% fetal bovine serum (FBS), 10 ng/ml epidermal growth factor, and 1 µg/ml hydrocortisone. 293 T cells were cultured in Dulbecco’s Modified Eagle’s medium (DMEM) (Mediatech) supplemented with 10% FBS and 1x pen-strep. Cells were grown in 10 cm plates and passaged before reaching confluency.

### Plasmids

The 3xFLAG N1ICD construct (Addgene #20183) was a gift from Raphael Kopan and contains amino acids Val1744 to Lys 2531 of the murine Notch1 intracellular domain with a 3xFLAG N-terminal tag^38^. The 3xFLAG N1ICD construct was subjected to site directed mutagenesis in order to create Y → F substitutions at Y1928 (Y1938 in humans), Y2064 (Y2074 in humans), Y2106 (Y2116 in humans), Y2135 (Y2145 in humans). This 3xFLAG N1ICD plasmid and a doxycycline inducible lenti viral destination vector, pCW57.1 (Addgene #41393, a gift from David Root) was used to construct a 3xFLAG N1ICD lentiviral expression vector. The integrin β3 construct (Addgene #27289) was a gift from Timothy Springer and contains human integrin β3 with a C-terminal myc-his tag^39^. The c-Src construct was a gift from William Schiemann and contains full length human c-Src with a C-terminal myc-his tag cloned into a pcDNA 3.1/myc-His B vector^40^. The constitutively active Src construct (Addgene #13660) was a gift from Joan Brugge and contains chicken Src with a Y527F mutation. The dominant negative Src construct (Addgene #13657) was a gift from Joan Brugge and Peter Howley and contain mouse Src K295R Y527F. The v-Src construct (Addgene #14578) was a gift from Joan Brugge and contains Src isolated from Rous sarcoma virus. The pcDNA 3.1 C-terminally myc-his tagged MAGP2 construct was previously described^41^. The Hes1 luciferase construct was a gift from Jan Jensen and consists of nucleotides −2553 to −201 relative to the murine Hes1 transcriptional start site while transcribing for firefly luciferase^42^. The Hes5 luciferase construct (Addgene #41724) was a gift from Ryoichiro Kageyama and Raphael Kopan and contains the murine Hes5 promoter (−800 to +73) relative to the Hes5 transcriptional start site while transcribing for firefly luciferase^42^. The 4xCSL luciferase construct (Addgene #41726) was a gift from Raphael Kopan and consists of 4 tandem repeats of the high affinity CSL binding sites (5′CGTGGGAA3′) while transcribing for firefly luciferase^38^. The BirA(R118G)-Ha destination vector (Addgene #53581) was a gift from Karl Kramer and was used to construct the N1ICD::BirA fusion and 2058–2161::BirA fusion which contains 2048–2151 of murine Notch1 (amino acids 2058–2161 in the human protein). The 2058–2161 peptide used in the *in vitro* kinase assays was constructed through gateway cloning into pET-DEST42. The MAML1 plasmid was a gift from Brandon J. White and contains human MAML1 with a myc tag cloned into a pCS2 backbone.

### N1ICD half-life analysis

In order to track N1ICD half-life, steady-state populations of HMEC-1 cells were treated with the γ-secretase inhibitor DAPT (10 µM) to specifically block S3 N1ICD cleavage/synthesis and cells were sacrificed after 0, 45, 90, 135 minutes DAPT treatment and blotted for N1ICD. Using densitometry, N1ICD half-life was calculated after 24 hours of treatment with SFK inhibitor (AZM475271) and DMSO control by normalizing signal intensity to β-actin housekeeping control then dividing the N1ICD amount after each time point by the starting amount.

### Proximity biotin ligation assays

In this BioID experiment, a mutated version of BirA biotin ligase (R118G BirA) was employed, which non-discriminately biotinylates proteins within close proximity allowing for streptavidin pull down of proximal proteins^43^. 293 T cells were *Trans*IT®-LT1 (Mirus) lipid transfected with either 2058–2161::BirA or N1ICD::BirA fusion constructs. 48 hours after transfection, cells were incubated in serum free media supplemented with 50 µM biotin for 6 hours before harvesting. Streptavidin magnetic beads (10 µL, New England BioLabs) were used to precipitate biotinylated species on a magnetic tube rack. Specific protein targets were detected using primary antibodies followed by membrane stripping before detection of overall biotinylated proteins. Biotinylated proteins were detected using horseradish peroxidase conjugated streptavidin (1:40,000) which was purchased from Thermo Scientific.

### *In vitro* c-Src kinase assays

A 14 kDa FLAG tagged region (amino acids 2058–2161) of the N1ICD protein was produced in *E*. *coli* and purified using FLAG affinity resin and dissolved in a TBS solution. In addition to a wild type form, two mutant forms of the peptide were created, Y2074F and Y2145F. An *in vitro* c-Src kinase assay was performed with purified GST tagged human c-Src (Sigma-Aldrich) using the manufacturer’s protocol. Briefly, the 14 kDa peptide was mixed into a cocktail containing 2 mM MOPS (pH 7.2), 1 mM glycerol 2-phosphate, 1.6 mM MgCl_2_, 1 mM MnCl_2_, 0.4 mM EGTA, 0.16 mM EDTA, 0.02 mM DTT, 16 ng/μL BSA, +/−0.05 mM ATP, +/−200 ng purified Src. Reaction volumes totaled 25 μL and were incubated for 15 minutes at 30 °C. Reactions were terminated by adding SDS page lysis buffer, vortexing, and boiling for 5 minutes and submitted to western blot analysis.

### Luciferase assays

HMEC-1 cells were seeded into 24-well plates at a density of 25,000 cells/well. 293 T cells were seeded into 24-well plates at a density of 50,000 cells/well. The following day, cells were transfected using *Trans*IT®-LT1 liposomes (Mirus). Cells were transfected with 100 ng/well Hes1 luciferase, Hes5 luciferase, or 4xCSL luciferase plasmids which produce luciferase in response to Notch pathway activation and 30 ng/well CMV-β-gal plasmid. Co-transfection of a CMV-Beta-Galactosidase construct was used to normalize data for transfection efficiency and potential cell death/proliferation. For AZM475271 treated cells, cells were treated with 10 µM AZM475271 for 24 h before being sacrificed on the next day. Cells were lysed 48 h after transfection using passive lysis buffer (Promega) and lysates were submitted to a luciferase reporter assay as per manufacturer’s protocol and analyzed using a Promega© Glomax Multi Detection System luminometer. Luciferase activity was normalized to Beta-Galactosidase activity and values were reported as fold change to control. All conditions were performed in triplicate for each independent experiment.

### Immunoprecipitations

Cells were washed twice with 1x PBS, scraped up on ice, and transferred to 1.7 ml tubes followed by pelleting by centrifugation whereupon supernatant was removed. Cells were lysed for 30 minutes in 500 µL of co-immunoprecipitation buffer (200 mM KCL, 25 mM Hepes, 1% NP-40, 20 mM NaF, 1 mM Na-orthovanadate, 0.2 mM EGTA, 1x protease arrest [G Biosciences], 1x phosphatase inhibitor cocktail II [Alfa Aesar], 20 µM nicotinamide, pH 7.5) and sonicated. Lysates were then subjected to centrifugation and whole cell lysate samples were generated using the 50 µL of the total lysate, to which 4x SDS page lysis buffer was added, followed by vortexing and boiling for 5 minutes. For immunoprecipitation of FLAG tagged proteins, the remaining 450 µL of lysate was incubated with 20 µL of anti-FLAG G1 affinity resin (GenScript) overnight on a tube rotator at 4 °C. After incubation, washing was performed to remove non-specific binding contaminates. Briefly, samples were centrifuged, supernatant removed, washed with 500 µL of co-immunoprecipitation buffer, and allowed to rotate at 4 °C on a tube rotator for 5 minutes. These steps were repeated three times, before a final centrifugation and removal of supernatant, after which 50 µL of 1x SDS page lysis buffer was added to the pellets before vortexing and boiling for 5 minutes.

### Western blotting

Cells were lysed in 1x SDS page lysis buffer and boiled for 5 minutes. Proteins were separated through SDS page on 6–15% polyacrylamide gels and blotted onto nitrocellulose membranes. Membranes were blocked in TBS-T (140 mM NaCL, 25 mM Tris-HCL, pH 7.4, 0.1% Tween-20) with 5% bovine serum albumin for 1 hour at room temperature. Membranes were incubated with primary antibody (1:250, 1:500, or 1:1000) overnight on a rotator at 4 °C. After incubation, membranes were washed 3 × 10 minutes in TBS-T before 1 hour incubation in secondary antibodies at room temperature. Horseradish peroxidase conjugated secondary antibodies were used at a concentration of 1:5000. After incubation with secondary antibodies, proteins were detected by enhanced chemiluminescence.

## Electronic supplementary material

Supplementary dataset 1
