# Supplementary material for: Src kinase phosphorylates Notch1 to inhibit MAML binding
Source: Sci Rep. 2018 Oct 19;8:15515. doi: 10.1038/s41598-018-33920-y (PMC6195628; doi:10.1038/s41598-018-33920-y)

SUPPLEMENTARY INFORMATION

Src kinase phosphorylates Notch1 to inhibit MAML binding

Bryce LaFoya[1], Jordan A. Munroe[2], Xinzhu Pu[3], & Allan R. Albig[1,2,#]

[1] Biomolecular Sciences PhD Program. Boise State University. Boise, ID 83725, USA

[2] Department of Biological Sciences. Boise State University. Boise, ID 83725, USA

[3] Biomolecular Research Center. Boise State University. Boise, ID 83725, USA

Running Title: Src kinase regulates Notch

Keywords: Notch, microenvironment, phosphorylation, Src, SFK, integrin, MAGP2, MAML, phosphotyrosine

# Address correspondence to Allan Albig. AllanAlbig@boisestate.edu

Supplementary Figure 1

Y2074: EGSYETAK, Y4-Phospho (79.96633 Da)

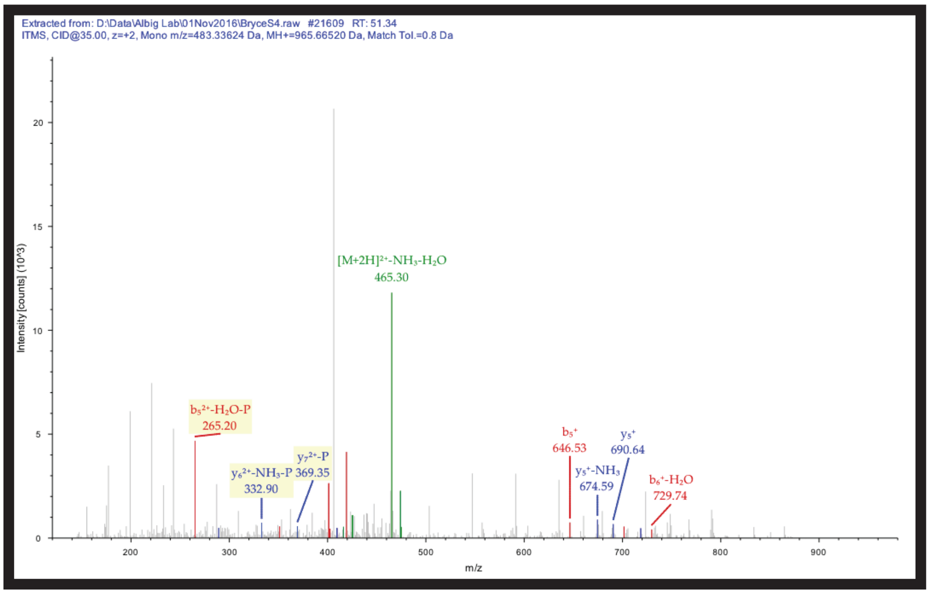

Y2116: MHHDIVRLLDEYNLVR, Y12-Phospho (79.96633 Da)

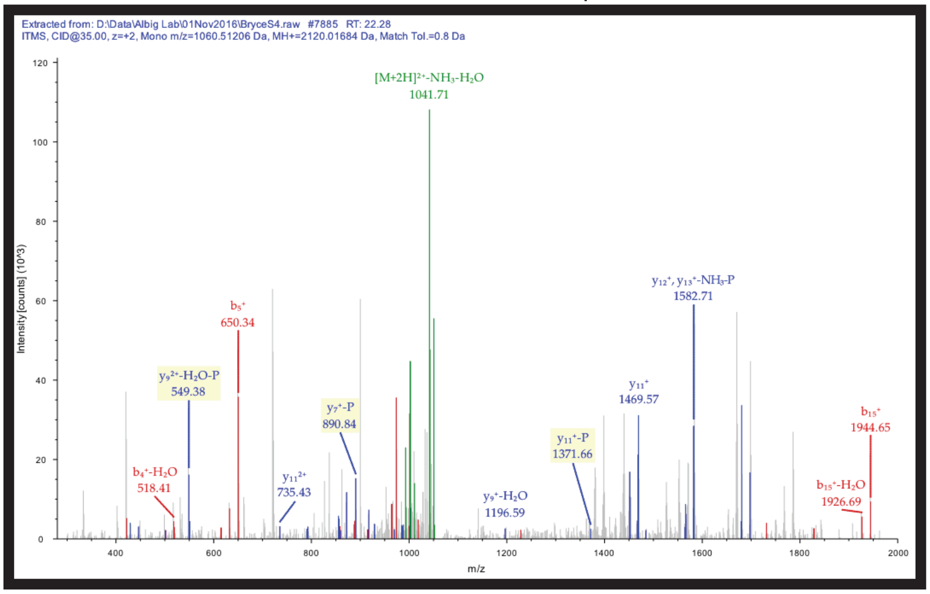

Y2145: SPQLHGTALGGTPTLSPTLCSPNGYLGNLK, Y25-Phospho (79.96633 Da)

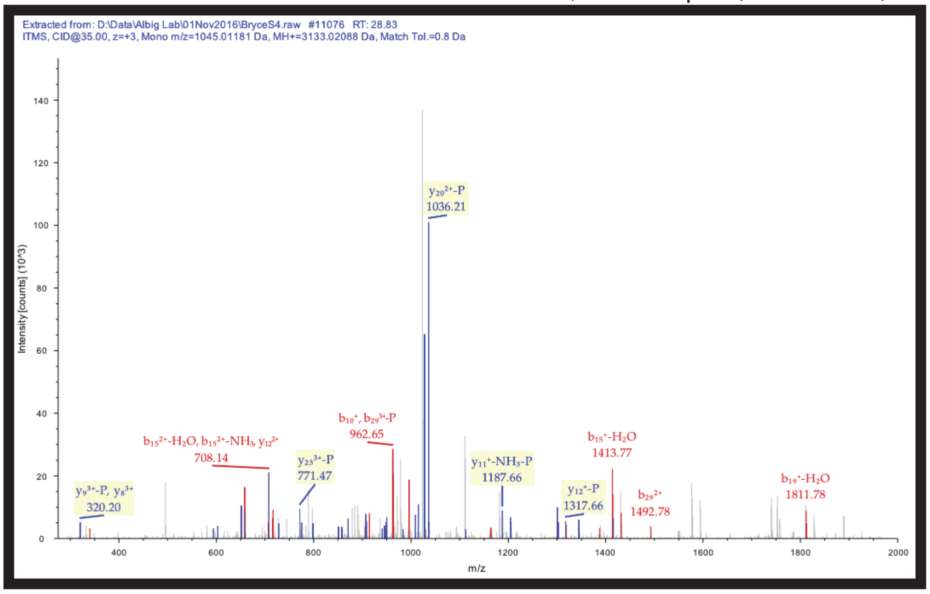

Supplementary Figure 2

Y1938: Sequence: TGETALHLAARYSR, Y12-Phospho (79.96633 Da)

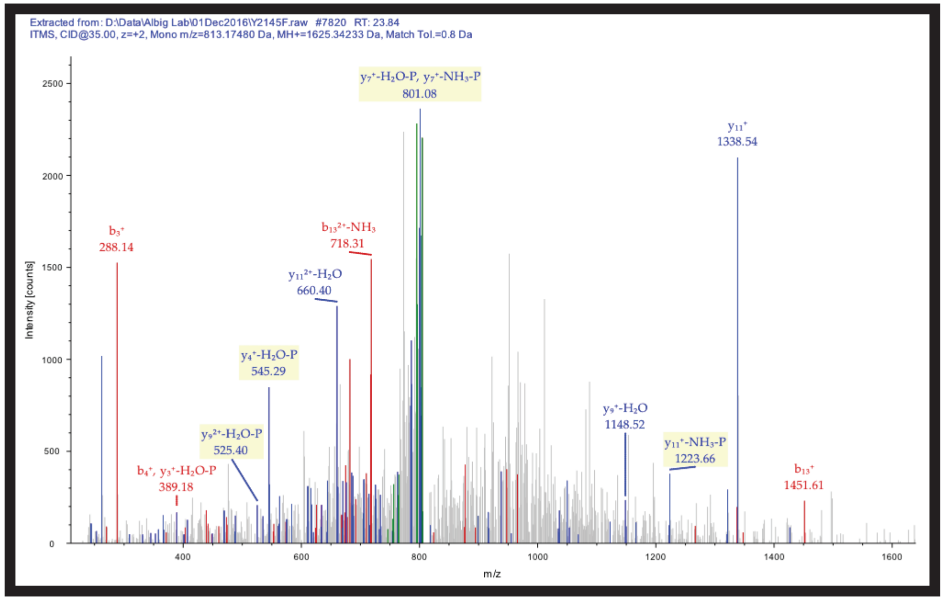

Y2074: DMQNNKEETPLFLAAREGSYETAK, Y20-Phospho (79.96633 Da)

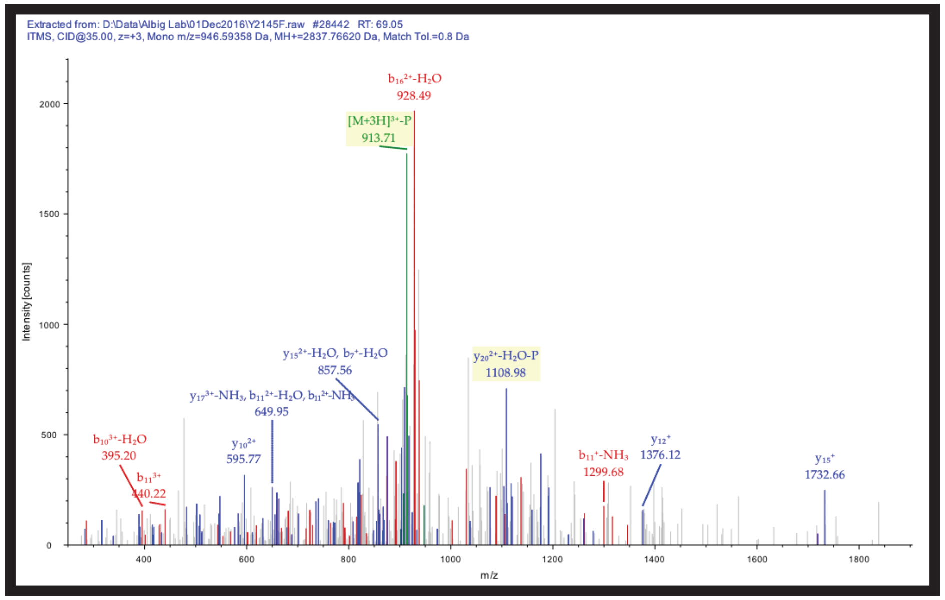

Y2116: LLDEYNLVRSPQLHGTALGGTPTLSPTLCSPNGYLGNLK, Y5-Phospho (79.96633 Da)

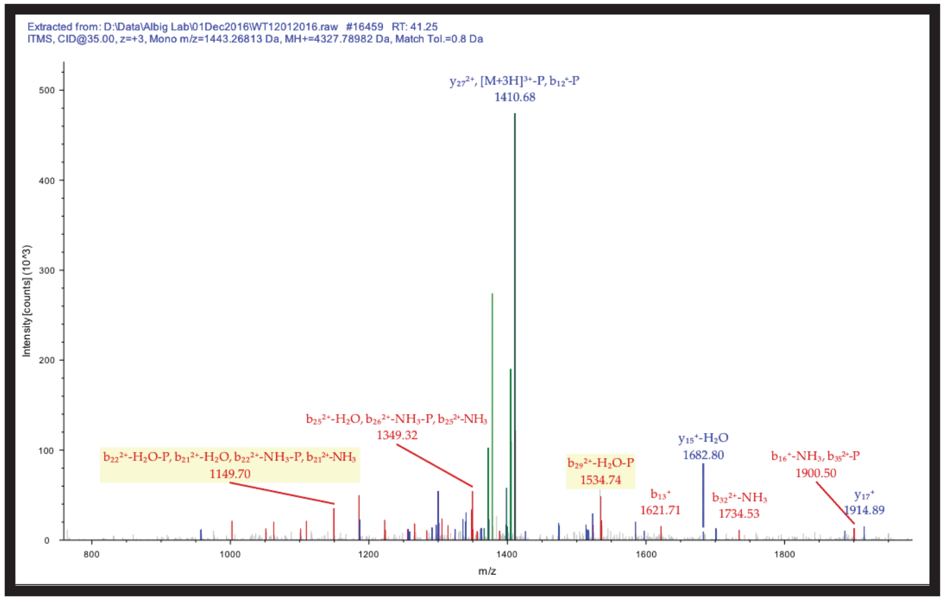

Y2145: SPQLHGTALGGTPTLSPTLCSPNGYLGNLK, Y25-Phospho , (79 .96633 Da)

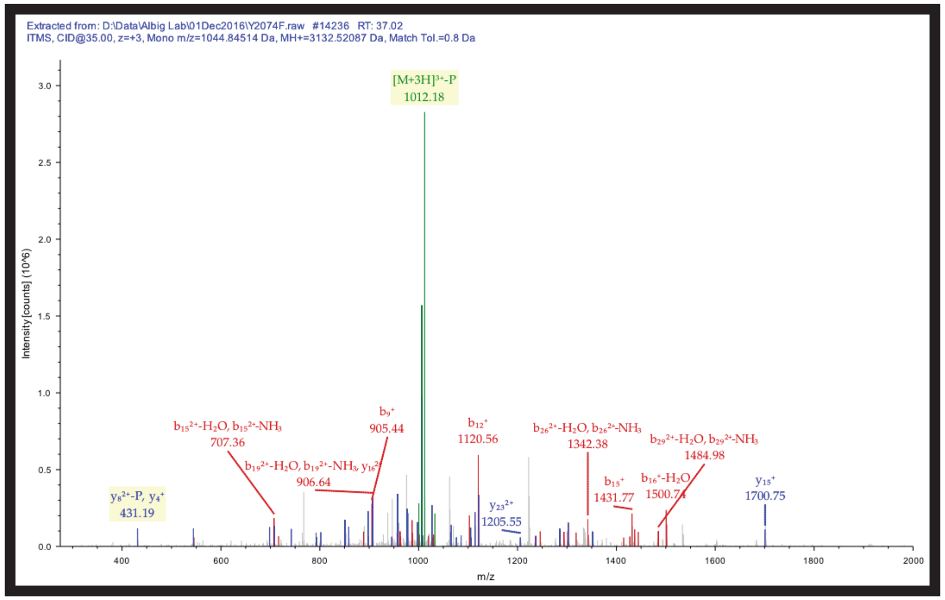

Fig. 1A

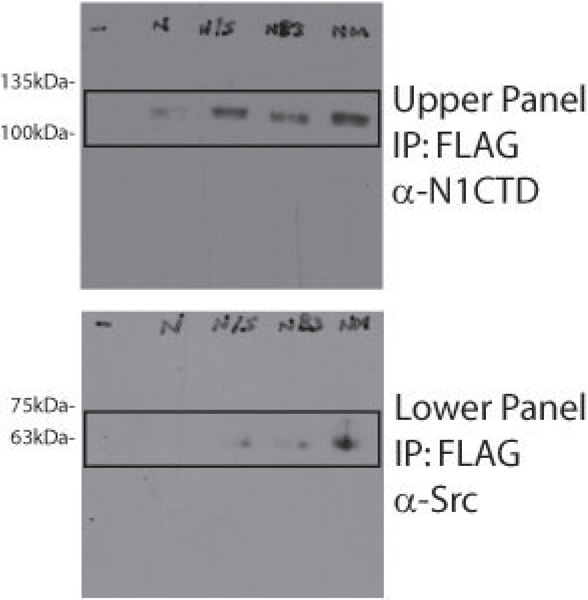
135kDa-
100kDa-
Upper Panel
IP: FLAG
α-N1CTD
75kDa-
63kDa-
Lower Panel
IP: FLAG
α-Src

Fig. 1C

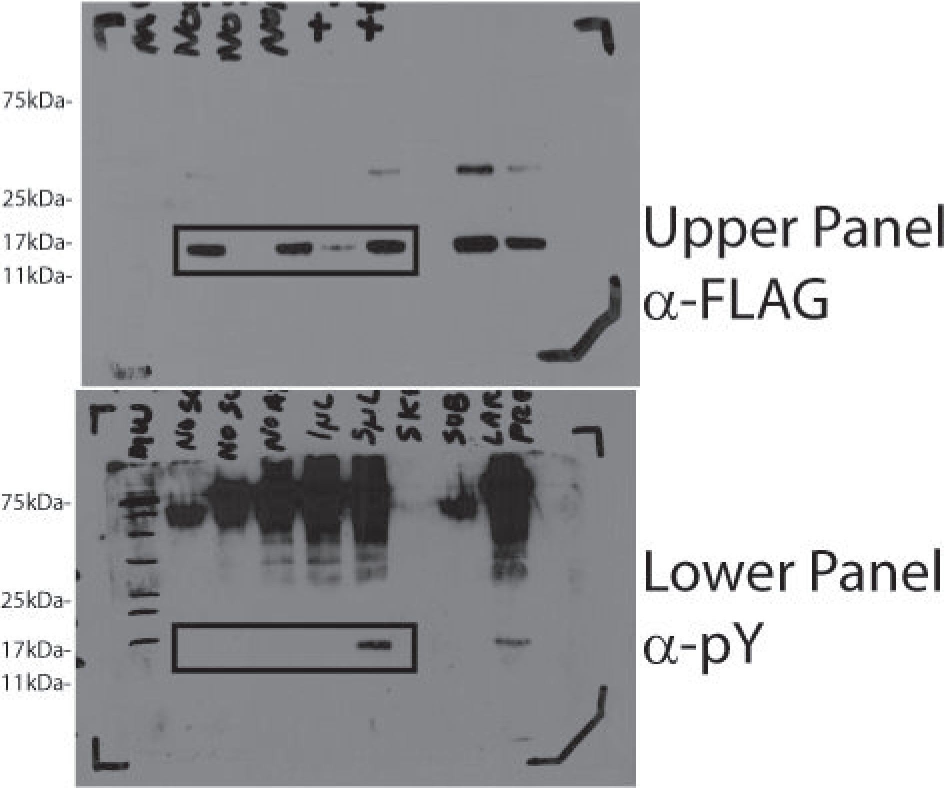
75kDa-
25kDa-
17kDa-
11kDa-
Upper Panel
α-FLAG
75kDa-
25kDa-
17kDa-
11kDa-
Lower Panel
α-pY

Fig. 1D

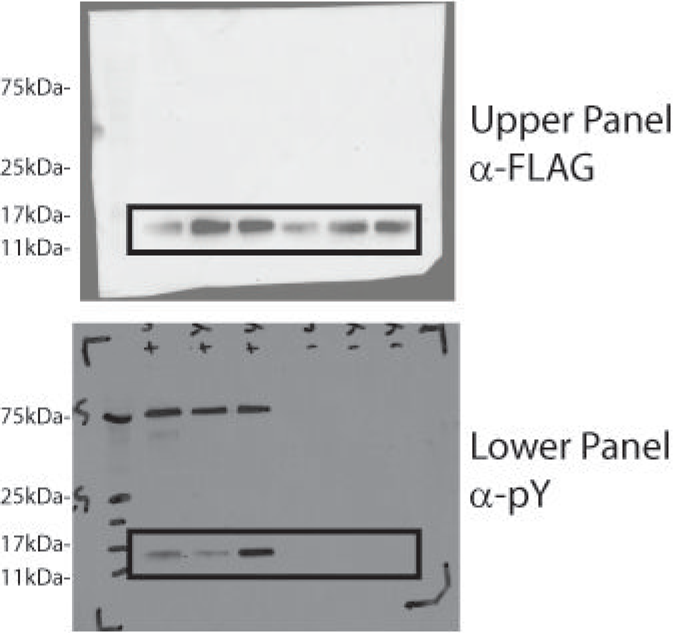
75kDa-
25kDa-
17kDa-
11kDa-
Upper Panel
α-FLAG
75kDa-
25kDa-
17kDa-
11kDa-
Lower Panel
α-pY

Fig. 2B

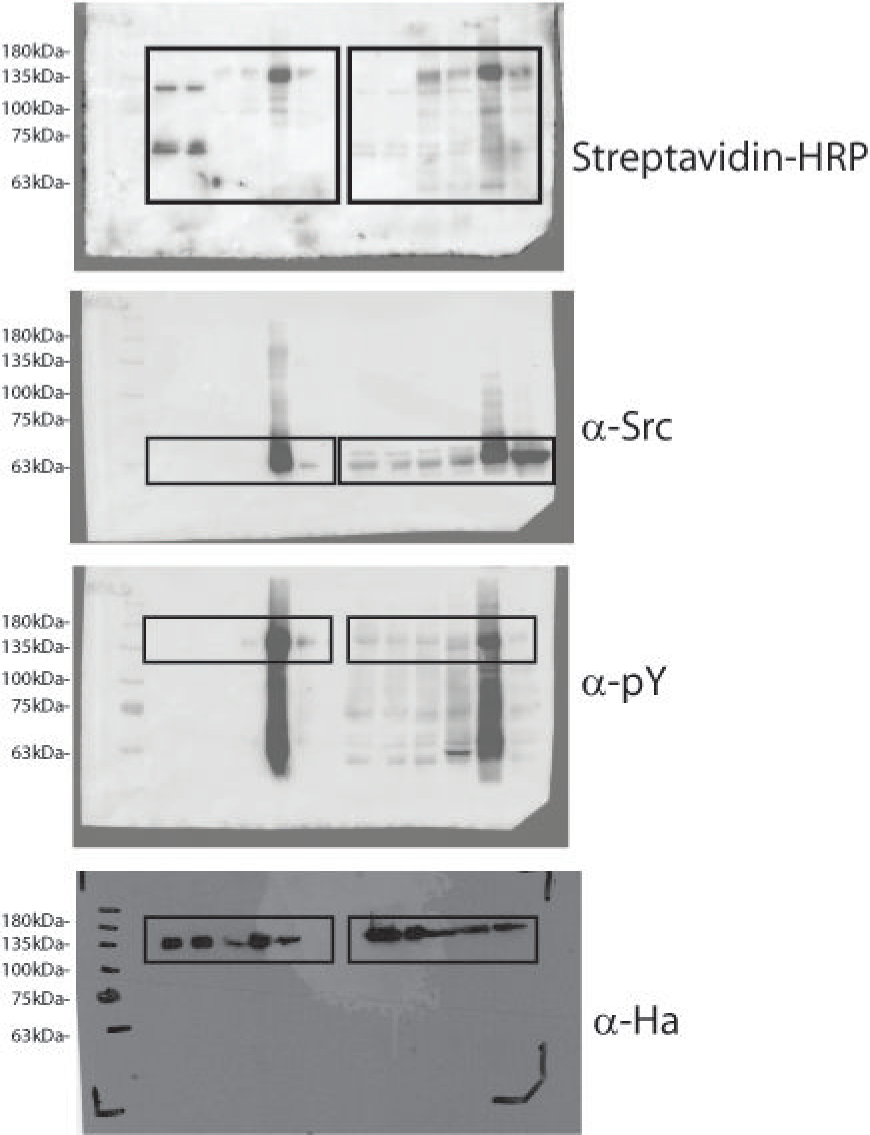
180kDa-
135kDa-
100kDa-
75kDa-
63kDa-
Streptavidin-HRP
180kDa-
135kDa-
100kDa-
75kDa-
63kDa-
α-Src
180kDa-
135kDa-
100kDa-
75kDa-
63kDa-
α-pY
180kDa-
135kDa-
100kDa-
75kDa-
63kDa-
α-Ha

Fig. 2D

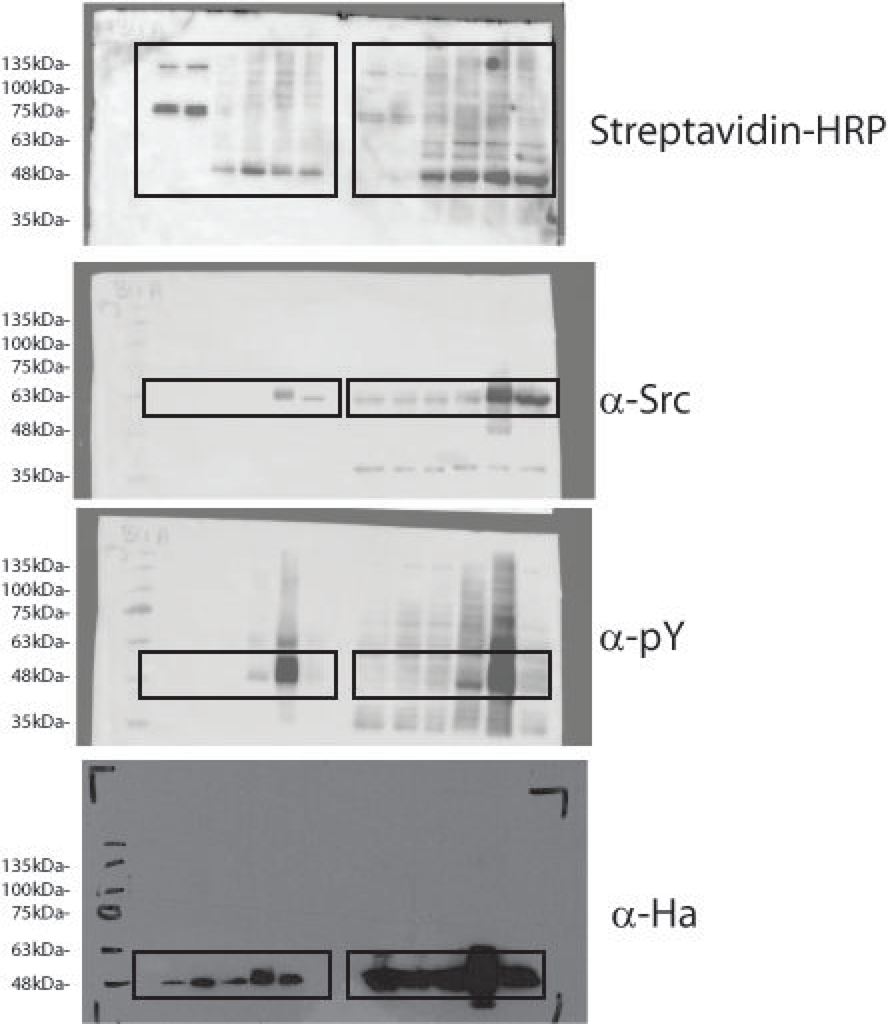

135kDa-
100kDa-
75kDa-
63kDa-
48kDa-
35kDa-
Streptavidin-HRP
135kDa-
100kDa-
75kDa-
63kDa-
48kDa-
35kDa-
α-Src
135kDa-
100kDa-
75kDa-
63kDa-
48kDa-
35kDa-
α-pY
135kDa-
100kDa-
75kDa-
63kDa-
48kDa-
α-Ha

Fig. 2E

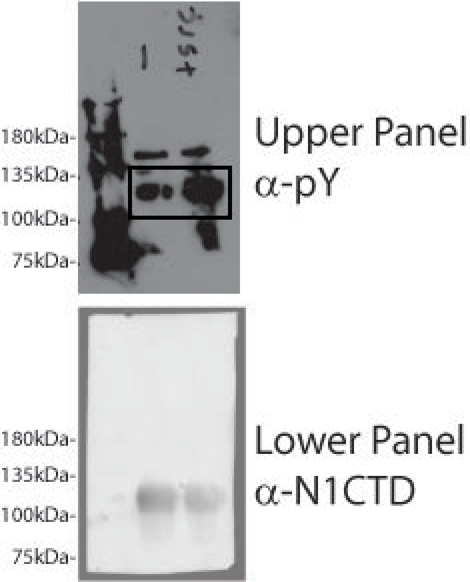
180kDa-
135kDa-
100kDa-
75kDa-
Upper Panel
α-pY
180kDa-
135kDa-
100kDa-
75kDa-
Lower Panel
α-N1CTD

Fig. 2F

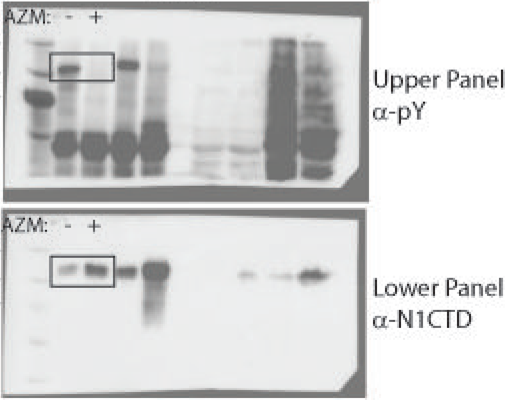

Fig. 2G

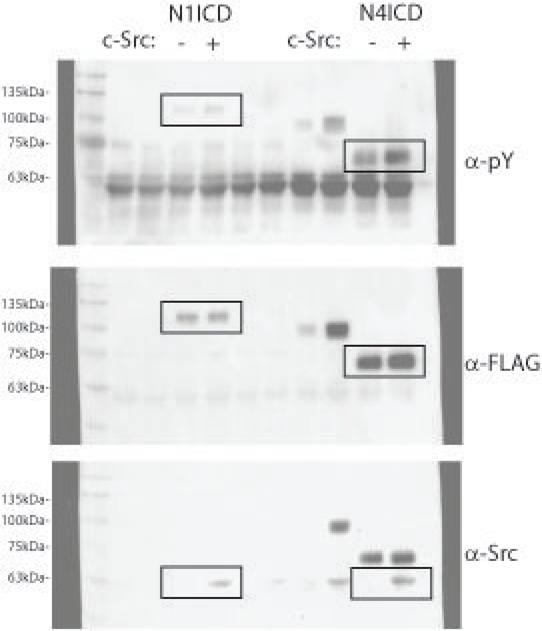
N1ICD
N4ICD
c-Src: - +
c-Src: - +
135kDa-
100kDa-
75kDa-
63kDa-
α-pY
α-FLAG
α-Src

Fig. 4B

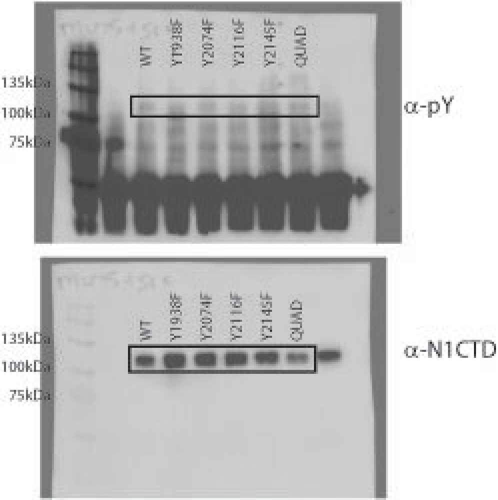
WT
Y1938F
Y2074F
Y2116F
Y2145F
QUAD
135kDa
100kDa
75kDa
α-pY
WT
Y1938F
Y2074F
Y2116F
Y2145F
QUAD
135kDa
100kDa
75kDa
α-N1CTD

Fig. 5A

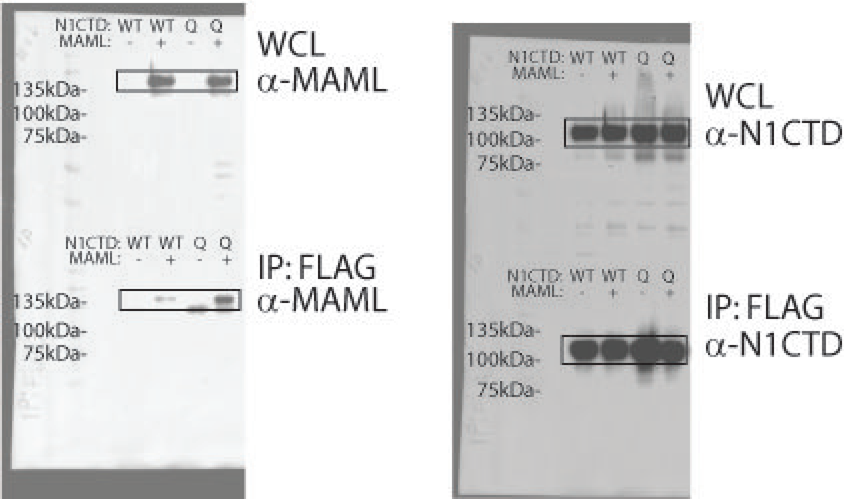

N1CTD: WT WT Q Q
MAML: - + - +
WCL
α-MAML
135kDa-
100kDa-
75kDa-
N1CTD: WT WT Q Q
MAML: - + - +
IP: FLAG
α-MAML
135kDa-
100kDa-
75kDa-
N1CTD: WT WT Q Q
MAML: - + - +
WCL
α-N1CTD
135kDa-
100kDa-
75kDa-
N1CTD: WT WT Q Q
MAML: - + - +
IP: FLAG
α-N1CTD
135kDa-
100kDa-
75kDa-

Fig. 5C

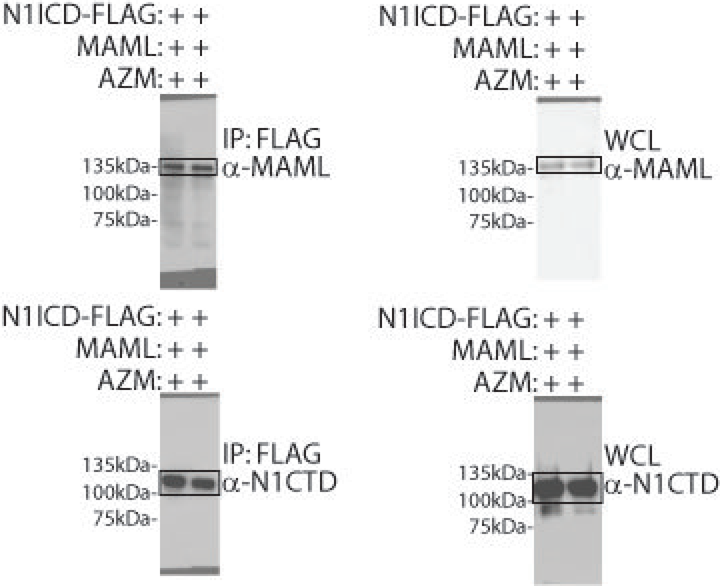
N1ICD-FLAG: + +
MAML: + +
AZM: + +
IP: FLAG
α-MAML
135kDa-
100kDa-
75kDa-
N1ICD-FLAG: + +
MAML: + +
AZM: + +
WCL
α-MAML
135kDa-
100kDa-
75kDa-
N1ICD-FLAG: + +
MAML: + +
AZM: + +
IP: FLAG
α-N1CTD
135kDa-
100kDa-
75kDa-
N1ICD-FLAG: + +
MAML: + +
AZM: + +
WCL
α-N1CTD
135kDa-
100kDa-
75kDa-

Fig. 5E

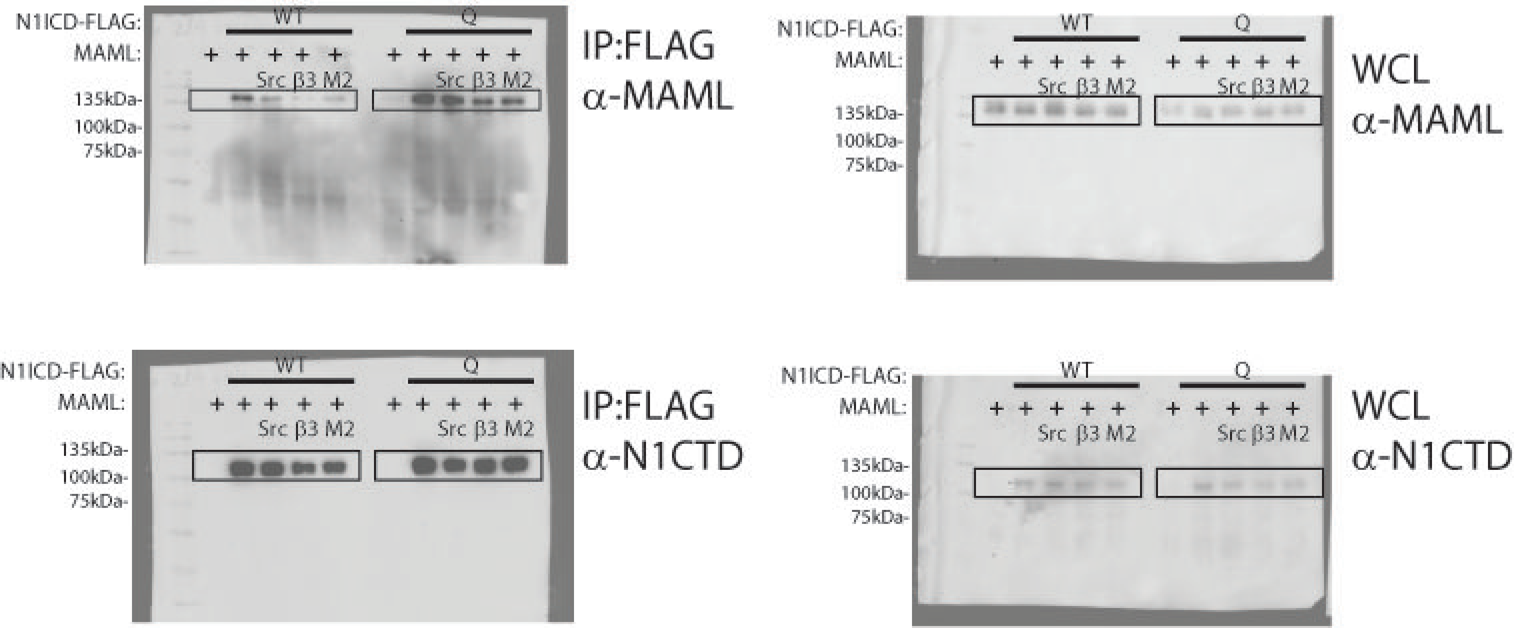

N1ICD-FLAG:
WT
Q
MAML:
+ + + + + + + + + +
Src β3 M2
Src β3 M2
135kDa-
100kDa-
75kDa-
IP:FLAG
α-MAML
WCL
α-MAML
IP:FLAG
α-N1CTD
WCL
α-N1CTD

Fig. 6B

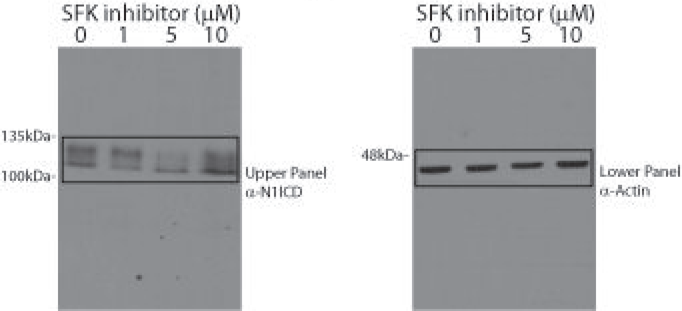
SFK inhibitor (μM)
0 1 5 10
135kDa-
100kDa-
Upper Panel
α-N1ICD
SFK inhibitor (μM)
0 1 5 10
48kDa-
Lower Panel
α-Actin

Fig. 6C

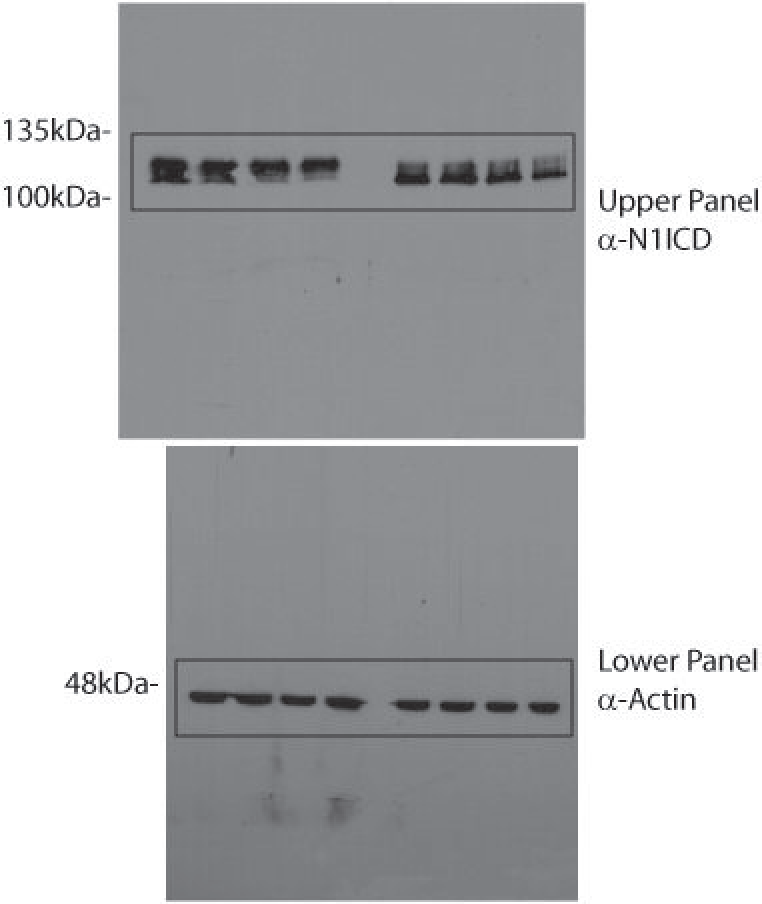

Fig. 6E

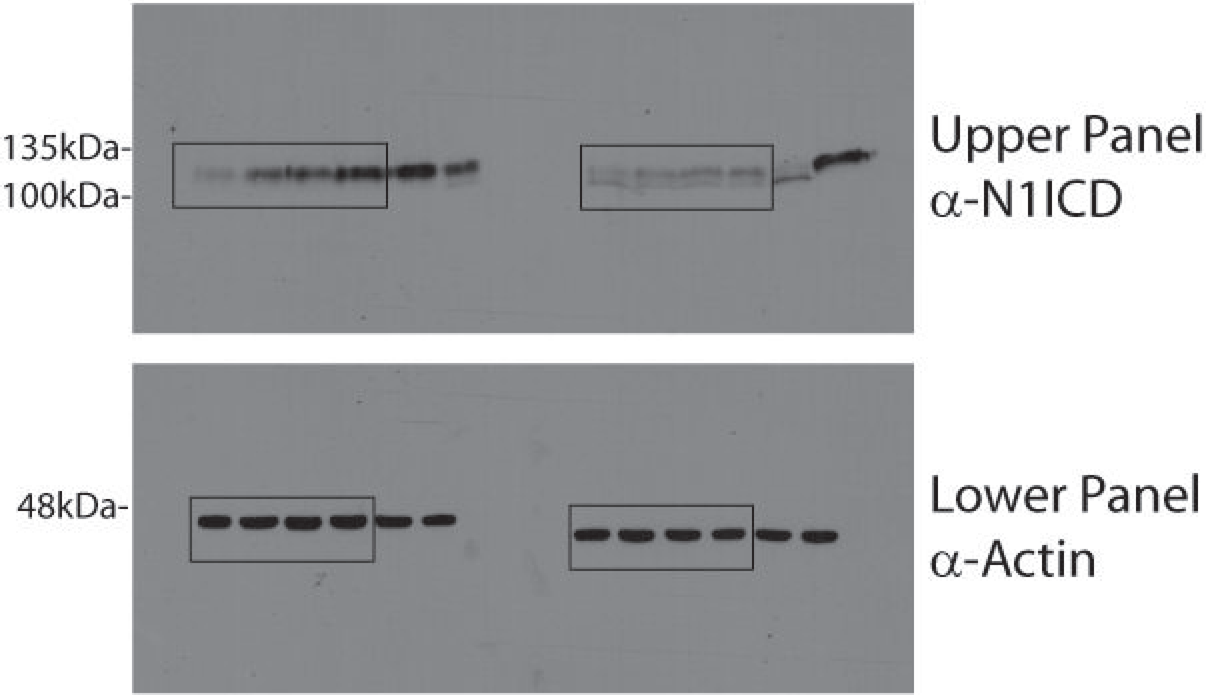

Supplement: Supplementary file 1 — Supplementary dataset 1 [file 41598_2018_33920_MOESM1_ESM.pdf]
